# Myofiber necroptosis promotes muscle stem cell proliferation via releasing Tenascin-C during regeneration

**DOI:** 10.1038/s41422-020-00393-6

**Published:** 2020-08-24

**Authors:** Shen’ao Zhou, Wei Zhang, Gaihong Cai, Yingzhe Ding, Caixia Wei, Sheng Li, Yu Yang, Jie Qin, Dan Liu, Hao Zhang, Xiexiang Shao, Jianhua Wang, Hongye Wang, Wenjun Yang, Huating Wang, She Chen, Ping Hu, Liming Sun

**Affiliations:** 1 State Key Laboratory of Cell Biology, CAS Center for Excellence in Molecular Cell Science, Shanghai Institute of Biochemistry and Cell Biology, Chinese Academy of Sciences, University of Chinese Academy of Sciences, Shanghai, 200031 China; 2 University of Chinese Academy of Sciences, Beijing, 100049 China; 3 National Institute of Biological Sciences, 7 Science Park Road, Zhongguancun Life Science Park, Beijing, 102206 China; 4 Li Ka Shing Institute of Health Sciences, The Chinese University of Hong Kong, Prince of Wales Hospital, New Territories, 999077 Hong Kong SAR China; 5 Department of Chemical Pathology, The Chinese University of Hong Kong, Prince of Wales Hospital, New Territories, 999077 Hong Kong SAR China; 6 Shuguang Hospital Affiliated to Shanghai University of Traditional Chinese Medicine, Shanghai, 201203 China; 7 Department of Orthopedic Surgery, Xin Hua Hospital Affiliated to Shanghai Jiao Tong University School of Medicine, Shanghai, 200092 China; 8 Department of Orthopaedics and Traumatology, The Chinese University of Hong Kong, Prince of Wales Hospital, New Territories, 999077 Hong Kong SAR China; 9 Tsinghua Institute of Multidisciplinary Biomedical Research, Tsinghua University, Beijing 102206, China; 10 Institute for Stem Cell and Regeneration, Chinese Academy of Sciences, Beijing, 100101 China; 11 Bioland Laboratory (Guangzhou Regenerative Medicine and Health Guangdong Laboratory), Guangzhou, Guangdong 510005 China; 12 Bio-Research Innovation Center, Shanghai Institute of Biochemistry and Cell Biology, Suzhou, Jiangsu 215121 China; 13 Shanghai Institute of Stem Cell Research and Clinical Translation, Shanghai, 200120 China

**Keywords:** Necroptosis, Muscle stem cells

## Abstract

Necroptosis, a form of programmed cell death, is characterized by the loss of membrane integrity and release of intracellular contents, the execution of which depends on the membrane-disrupting activity of the Mixed Lineage Kinase Domain-Like protein (MLKL) upon its phosphorylation. Here we found myofibers committed MLKL-dependent necroptosis after muscle injury. Either pharmacological inhibition of the necroptosis upstream kinase Receptor Interacting Protein Kinases 1 (RIPK1) or genetic ablation of MLKL expression in myofibers led to significant muscle regeneration defects. By releasing factors into the muscle stem cell (MuSC) microenvironment, necroptotic myofibers facilitated muscle regeneration. Tenascin-C (TNC), released by necroptotic myofibers, was found to be critical for MuSC proliferation. The temporary expression of TNC in myofibers is tightly controlled by necroptosis; the extracellular release of TNC depends on necroptotic membrane rupture. TNC directly activated EGF receptor (EGFR) signaling pathway in MuSCs through its N-terminus assembly domain together with the EGF-like domain. These findings indicate that necroptosis plays a key role in promoting MuSC proliferation to facilitate muscle regeneration.

## Introduction

Necroptosis is tightly regulated by the kinase activities of RIPK1 and Receptor Interacting Protein Kinase 3 (RIPK3).^1–4^ Upon necroptosis induction, RIPK1 binds with RIPK3 and form amyloidal death complex through their RIP homotypic interaction motif (RHIM) domains to activate their kinase activities.^5,6^ The amyloidal RIPK1-RIPK3 complex propagates death signal to a downstream effector protein MLKL. MLKL was found to be specifically required for necroptosis execution.^7,8^ Activated RIPK3 recruits and phosphorylates MLKL (on Ser345 of mouse MLKL; on Thr357/Ser358 of human MLKL), which releases the auto-inhibition of MLKL and enable its oligomerization through its brace region.^9,10^ The anti-phospho-MLKL antibody was widely used on sections as the biomarker to detect necroptosis happening in vivo.^11–17^ Following phosphorylation and oligomerization, the N-terminus MLKL directly binds with phosphatidylinositol phosphates (PIPs), which enables it to translocate to membrane compartments, which leads to membrane rupture and cellular contents release.^7–10,18–20^ Both *Mlkl-* and *Ripk3*-deficient mice were demonstrated to be developmentally normal.^21–23^ It has been widely reported that MLKL-mediated necroptosis plays an essential role in driving inflammation by releasing damage-associated molecular patterns (DAMPs).^24–27^ Therefore, necroptosis has been considered to be detrimental in vivo due to its pro-inflammatory features. However, whether necroptotic cell-released factors play beneficial roles under pathophysiological conditions is not clear.

Skeletal muscle has a remarkable capacity for regeneration after injury. Muscle regeneration is a highly coordinated process. Decades of pathological observations have reached a consensus that muscle repair usually starts with myofiber degeneration, which morphologically resembles necrosis.^28,29^ Whether this morphological resemblance of myofiber necrosis reflects the nature of necroptosis is not known. How myofiber necrosis contributes to tissue repair is also not clear.

Following muscle injury, the regeneration process greatly relies on the activation, proliferation and differentiation of muscle stem cells (MuSCs; also known as muscle satellite cells).^30–32^ In healthy unstressed adult muscles, MuSCs maintain a mitotically quiescent state, while acute injury quickly triggers MuSCs entry into cell cycle.^33–35^ Pax7, a transcription factor specifically expressed in MuSCs, is widely taken as the canonical biomarker for MuSCs.^36–38^ Following MuSC proliferation, a hierarchical expression of the myogenic regulatory factors (MRFs) Myf5, MyoD, myogenin (MyoG), and MRF4 drives the differentiation of MuSCs into new myofibers.^39,40^ The dynamic interplays between intrinsic factors within MuSCs and the extrinsic factors constituting the MuSC niche/microenvironment^41^ are tightly regulated during muscle regeneration. MuSCs are located beneath the surrounding basal lamina and outside the myofiber plasma membrane, in close proximity to myofibers. The myofibers naturally provide the primary components of the MuSC niche/microenvironment. Whether necroptosis contributes to modulating the composition of MuSC microenvironment has not been explored.

Here, we found increased expression of necroptosis components and phosphorylation of MLKL in myofibers upon injury. Necroptosis-deficient mice displayed muscle regeneration defects due to restrained MuSC proliferation during repair. Following a biochemical purification scheme, we identified Tenascin-C (TNC), released by necroptotic myofibers, as a key factor to promote MuSC proliferation. Further studies revealed that TNC was highly expressed in necroptotic myofibers and released to the microenvironment of MuSCs after injury. With its assembly domain combining with EGF-like domain, TNC served as an EGF mimic to stimulate MuSC proliferation to promote muscle regeneration.

## Results

### Necroptosis-deficient mice exhibit muscle regeneration defects

To explore the function of RIPK1/RIPK3/MLKL-axis dependent necroptosis in muscle regeneration, we generated necroptosis-deficient mice by knocking out the gene of MLKL using the CRISPR/Cas9 system (Supplementary information, Fig. S1a–c). Consistent with the previous report,^23^ these mice grew and developed normally. No obvious defects of skeletal muscle were observed in *Mlkl*^−/−^ mice, as their body weight and average myofiber size are the same as WT mice (Supplementary information, Fig. S1d, e), suggesting that necroptosis is not required for muscle development.

**Fig. 1:**
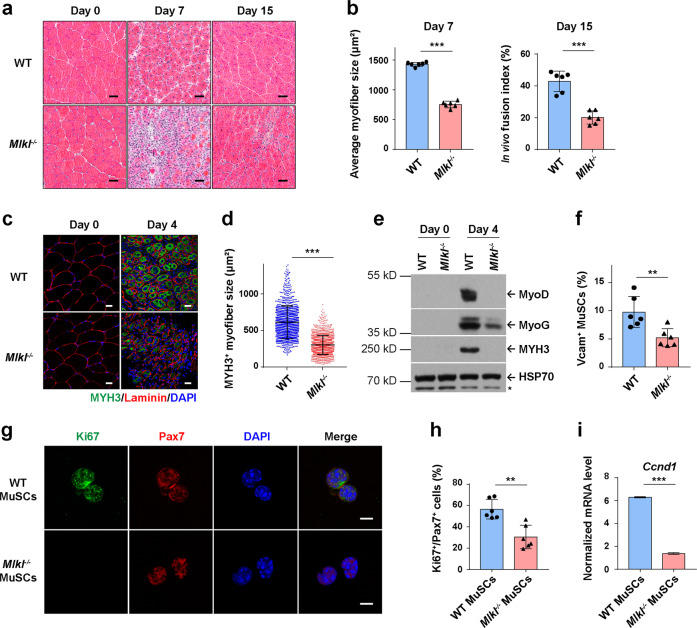
Necroptosis-deficient mice exhibit muscle regeneration defects. **a** Representative of H&E staining of TA muscle cross-sections from both uninjured (Day 0) and injured (7 and 15 days after CTX injection) WT and *Mlkl*^−/−^ mice. Scale bars, 50 μm. **b** Quantification of myofiber sizes from cross-sectional areas (CSAs) of injured mice (7 days after CTX injection) and myofibers with multiple central nuclei out of total cells with central nuclei (in vivo fusion index) 15 days post CTX injection. 3 different views were counted for each mouse. The data are expressed as the means ± SD. *n* = 6 each for WT and *Mlkl*^−/−^ mice. **c** Immunofluorescence staining of MYH3 (green) and Laminin (red) in TA muscle cross sections from both uninjured (Day 0) and injured (4 days after CTX injection) mice. Nuclei were identified by staining with DAPI. Scale bars, 25 μm. **d** Quantification of the MYH3^+^ myofiber sizes from CSAs of injured mice (4 days after CTX injection, as representatively shown in **c**). The sizes of 200 adjacent MYH3^+^ myofibers were measured for each mouse. Each dot represents an individual myofiber. The data are expressed as the means ± SD. *n* = 6 each for WT and *Mlkl*^−/−^ mice. **e** Immunoblotting analysis of MyoD, MyoG, and MYH3 expression in uninjured (Day 0) or injured (4 days after CTX injection) TA muscles using antibodies as indicated. Whole TA muscle lysates were exacted, as described in Materials and Methods, from 3 mice and pooled together for each condition. HSP70 serves as the loading control. The asterisk (*) denotes the non-specific band. Experiments were repeated independently for three times. **f** Quantification of the MuSCs in injured TA muscles (3 days after CTX injection) by FACS analysis. MuSCs were isolated as described in Materials and Methods. The histogram represents the percentage of MuSCs (PI^−^CD11b^−^CD31^−^CD45^−^Sca1^−^Vcam^+^ population) out of the total digested mono-nucleus cells. Each dot represents an individual mouse. The data are expressed as the means ± SD. *n* = 6 each for WT and *Mlkl*^−/−^ mice. **g** Immunofluorescence staining of Ki67 (green) and Pax7 (red) in freshly isolated MuSCs (3 days after CTX injection). MuSCs were isolated by FACS and fixed on PDL/Collagen-I pre-coated coverslip, as described in Materials and Methods, followed by immunofluorescence staining using the antibodies as indicated. Nuclei were identified by staining with DAPI. Scale bars, 5 μm. **h** Quantification of the Ki67^+^/Pax7^+^ cells as shown in **g**. MuSCs isolated from 3 mice were pooled together for immunofluorescence staining in each group. The histogram represents the percentage of Ki67^+^/Pax7^+^ cells out of 200 Pax7^+^cells per genotype. The data are expressed as the means ± SD of 6 images. **i** qRT-PCR analysis of *Ccnd1* mRNA level in MuSCs isolated from injured mice (3 days after CTX injection). The mRNA level of *Gapdh* was used as the internal control. MuSCs isolated from 3 mice were pooled together for qRT-PCR analysis. The data are expressed as the means ± SD of 3 technical repeats. *P* values for **b**, **d**, and **f** were determined by unpaired two-tailed *t*-test; *P* values for **h** and **i** were determined by unpaired two-tailed *t*-test with Welch’s correction. **P < 0.01; ***P < 0.005. CTX, cardiotoxin; TA, tibialis anterior.

Next we induced muscle injury by Cardiotoxin (CTX) injection as reported^42–45^ in both WT and *Mlkl*^−/−^ mice. After injury, the tibialis anterior (TA) muscles were harvested to investigate the muscle regeneration progression. Hematoxylin & eosin (HE) staining of the TA muscle cryo-sections was performed. On Day 7 post CTX injection, WT mice showed massive regenerated myofibers with central nuclei; in contrast, *Mlkl*^−/−^ mice displayed disorganized muscle structures and ∼50% reduction in average myofiber size; and the fusion index of myofibers in *Mlkl*^−/−^ mice was also reduced on 15 dpi (days post injury) (Fig. 1a, b). To confirm whether necroptosis deficiency causes muscle regeneration defects, we applied necroptosis inhibitor, Nec-1s,^46^ to the TA muscles in WT mice before and after CTX injection. As a control, the apoptosis inhibitor z-VAD-fmk was also tested in parallel. The Nec-1s-treated TA muscles displayed obvious defects in muscle regeneration, resembling what have been observed in *Mlkl*^−/−^ mice, while apoptosis inhibitor z-VAD-fmk treatment did not cause any obvious regeneration defects (Supplementary information, Fig. S1f, g). Further, in line with the observations in *Mlkl*^−/−^ mice, we found *Ripk3*^−/−^ mice also displayed similar muscle regeneration defects (Supplementary information, Fig. S1h–j). Immunofluorescence (IF) staining of MYH3, a marker for the nascent myofibers,^39,40^ showed significantly reduced size of the regenerated myofibers in *Mlkl*^−/−^ mice compared with that of WT mice (Fig. 1c, d). Consistently, expression level of the key myogenic factor MyoD and MyoG was significantly lower in regenerating muscles of *Mlkl*^−/−^ compared with WT littermates (Fig. 1e). Together, these results suggest that necroptosis is required for proper muscle regeneration.

MuSCs are the major cell source for muscle regeneration. Upon injury, MuSCs are activated to undergo fast proliferation.^33–35^ Before injury, necroptosis deficiency did not affect the number and location of MuSCs in *Mlkl*^−/−^ mice (Supplementary information, Fig. S1k–m). In contrast, after CTX injection, the number of MuSCs was significantly lower in *Mlkl*^−/−^ muscles than WT muscles (Fig. 1f), as quantified by FACS analysis as previously described.^47,48^ Co-immunofluorescence staining of Ki67 with Pax7 showed reduced number of proliferating MuSCs in *Mlkl*^−/−^ mice than in WT mice (Fig. 1g, h). Quantitative reverse transcription PCR (RT-qPCR) analysis showed a significant lower level of the *Ccnd1* (encodes Cyclin D1) mRNA in MuSCs isolated from injured *Mlkl*^−/−^ TA muscles (Fig. 1i). These results demonstrate that *Mlkl*^−/−^ MuSCs display lower proliferation ability. However, when we examined the proliferation and differentiation ability of the freshly isolated MuSCs, as previously described,^45^ no obvious difference was observed between *Mlkl*^−/−^ and WT mice (Supplementary information, Fig. S1n–p). These results suggest necroptosis deficiency causes MuSC microenvironmental defects after injury.

### Myofiber-specific necroptosis is required for MuSC proliferation upon muscle injury

**Fig. 2:**
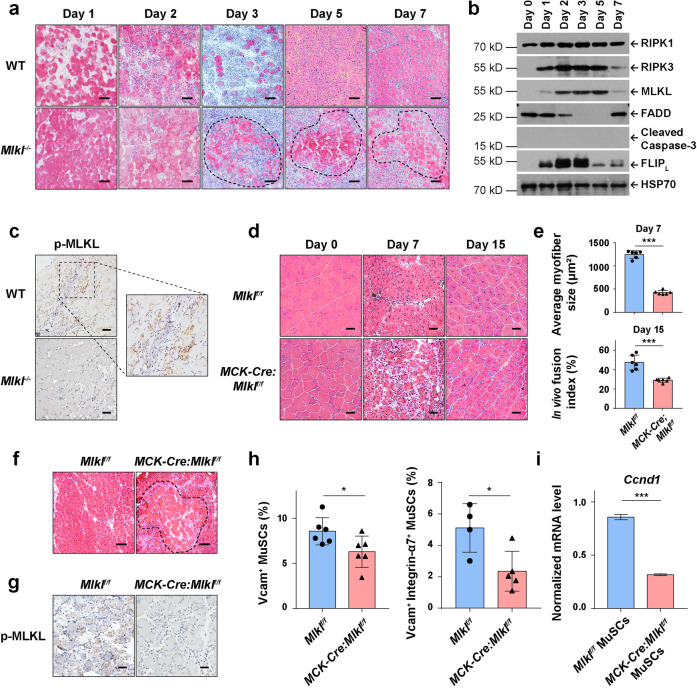
Necroptotic myofibers are indispensable for post injury MuSC proliferation. **a** Representative H&E staining of TA muscle cross-sections from injured mice at different time points (0, 1, 2, 3, 5, and 7 days after CTX injection, respectively). The continuous dotted lines delineate the borders of death-resistant myofibers. Scale bars, 100 μm. **b** Immunoblotting analysis of RIPK1, RIPK3, MLKL, FADD, Cleaved Caspase-3, and FLIP_L_ expression in myofibers at different time points as indicated. Myofibers were purified from TA muscles as described in Materials and Methods. Whole myofiber lysates were exacted from 3 mice and pooled together for each condition. HSP70 serves as the loading control. Experiments were repeated independently for more than three times. **c** Representative immunohistochemical staining of p-MLKL in TA muscle cross sections from injured mice (2 days after CTX injection). The signals of p-MLKL appear brown in sections counter-stained with hematoxylin (blue). Experiments were repeated independently for more than three times. Scale bars, 50 μm. **d** Representative of H&E staining of TA muscle cross-sections from both uninjured (Day 0) and injured (7 and 15 days after CTX injection) *Mlkl*^*f/f*^ and *MCK-Cre:Mlkl*^*f/f*^ mice. Scale bars, 50 μm. **e** Quantification of myofiber sizes from cross-sectional areas (CSAs) of injured mice (7 days after CTX injection) and myofibers with multiple central nuclei out of total cells with central nuclei (in vivo fusion index) 15 days post CTX injection. 3 different views were counted for each mouse. The data are expressed as the means ± SD. *n* = 6 each for *Mlkl*^*f/f*^ and *MCK-Cre:Mlkl*^*f/f*^ mice. **f** Representative H&E staining of TA muscle cross-sections from injured (7 days after CTX injection) *Mlkl*^*f/f*^ and *MCK-Cre:Mlkl*^*f/f*^ mice. The continuous dotted line delineates the borders of cell death-resistant myofibers. Scale bars, 100 μm. **g** Representative immunohistochemical staining of p-MLKL in TA muscle cross sections from injured (2 days after CTX injection) *Mlkl*^*f/f*^ and *MCK-Cre:Mlkl*^*f/f*^ mice. The signals of p-MLKL appear brown in sections counter-stained with hematoxylin (blue). Experiments were repeated independently for more than three times. Scale bars, 50 μm. **h** Quantification of the MuSCs in injured TA muscles (3 days after CTX injection) by FACS analysis. MuSCs were isolated as described in Materials and Methods. Histogram represents percentage of MuSCs (PI^-^CD11b^-^CD31^-^CD45^-^Sca1^-^Vcam^+^ population, and 7-AAD^-^CD11b^-^CD31^-^CD45^-^Sca1^-^Vcam^+^Integrin-α7^+^ population) out of the total digested mono-nucleus cells. The data are expressed as the means ± SD. Left panel: *n* = 6 each for *Mlkl*^*f/f*^ and *MCK-Cre:Mlkl*^*f/f*^ mice. Right panel: *n* = 4 for *Mlkl*^*f/f*^ and *n* = 5 for *MCK-Cre:Mlkl*^*f/f*^ mice. **i** qRT-PCR analysis of *Ccnd1* mRNA level in MuSCs isolated from injured mice (3 days after CTX injection). The mRNA level of *Gapdh* was used as the internal control. MuSCs isolated from 3 mice were pooled together for qRT-PCR analysis. The data are expressed as the means ± SD of 3 technical repeats. *P* values for **e** and **i** were determined by unpaired two-tailed *t*-test; *P* values for **h** were determined by unpaired two-tailed *t*-test with Welch’s correction. *P < 0.05; ***P < 0.005.

We next set out to identify the cell type going through necroptosis after muscle injury. During muscle regeneration, massive death-resistant myofibers clustered at the injury site in *Mlkl*^−/−^ mice but not in WT mice (Fig. 2a). Based on this observation, we hypothesized that the myofibers may die via necroptosis. To test this hypothesis, we isolated the myofibers throughout the repair process and analyzed the expression patterns of both the apoptosis and necroptosis essential components by immunoblotting. Consistent with the previous report,^23,49^ RIPK3 and MLKL were hardly detectable in uninjured muscle (Fig. 2b, Day 0). Interestingly, the expression level of both RIPK3 and MLKL started to dramatically increase from post injury Day 1, and faded on Day 7 (Fig. 2b). In contrast, the expression of apoptosis protein FADD was downregulated; no Caspase-3 activation was detected; the anti-apoptosis protein c-FLIP_L_ was upregulated along with the rising of RIPK3/MLKL expression (Fig. 2b). These data indicate necroptosis might be activated in myofibers after muscle injury, while apoptosis was not activated. We next examined MLKL phosphorylation, the key activating event of necroptosis,^7,10^ in injured TA muscles by immunohistochemistry staining using anti-phospho-MLKL antibody. Strikingly, the phospho-MLKL (p-MLKL) signals were detected in WT but not *Mlkl*^−/−^ TA sections after injury (Fig. 2c). Consistently, after necroptosis inhibitor Nec-1s treatment, the death-resistant myofibers were accumulated and p-MLKL signals were abolished in WT mice (Supplementary information, Fig. S2a, b). These results suggest that myofibers committed MLKL-dependent necroptosis after muscle injury.

Next we generated skeletal myofiber-specific *Mlkl*-deleted mice, the *MCK-Cre:Mlkl*^*f/f*^ mice (Supplementary information, Fig. 2c–f). After CTX injection. We found that the *MCK-Cre:Mlkl*^*f/f*^ mice phenocopy the defects of muscle regeneration of the constitutive *Mlkl*-knockout mice: reduced average nascent myofiber size on 7 dpi and reduced fusion index on 15 dpi (Fig. 2d, e); clustered death-resistant myofibers (Fig. 2f) and abolished p-MLKL signals (Fig. 2g); declined number of MuSCs and lower *Ccnd1* mRNA level in MuSCs during repair (Fig. 2h, i). In vivo propidium iodide (PI) staining showed that *MCK-Cre:Mlkl*^*f/f*^ mice have significantly decreased number of necrotic dying cells after muscle injury (Supplementary information, Fig. S2g, h). In vivo EdU staining showed the MuSC proliferation was attenuated in *MCK-Cre:Mlkl*^*f/f*^ mice after injury (Supplementary information, Fig. S2i–k). In addition, we utilized BaCl_2_ injection, another widely-used muscle injury model,^42,50,51^ confirming the muscle regeneration defects of *MCK-Cre:Mlkl*^*f/f*^ mice (Supplementary information, Fig. S2l–o). Taken together, these results demonstrate that necroptosis of myofibers is required for MuSC proliferation in vivo.

### Necroptotic muscle cells release factors to promote MuSC proliferation

**Fig. 3:**
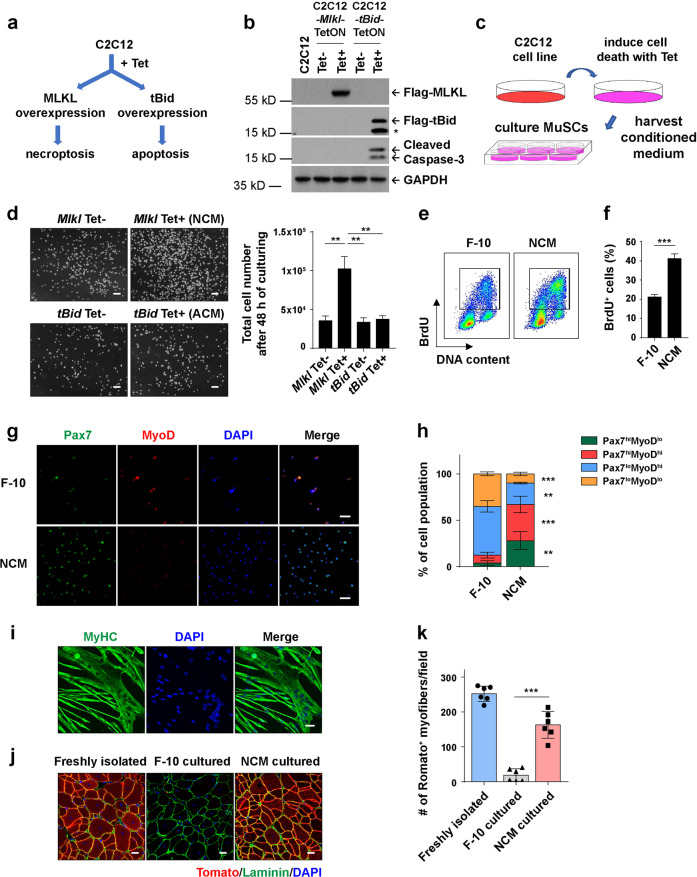
Necroptotic muscle cells release factors to promote MuSC proliferation. **a** Schematics of generating stable C2C12 cell lines that are capable of Tet-induced necroptosis or apoptosis, through overexpressing MLKL or tBid, respectively. **b** Immunoblotting analysis of MLKL-Flag, tBid-Flag, and Cleaved Caspase-3 expression in cell death-inducible C2C12 cell lines with or without tetracycline treatment. C2C12-*Mlkl*-TetON cells were treated with 1 μg/mL tetracycline for 12 h to induce necroptosis, and C2C12-*tBid*-TetON cells were treated with 1 μg/mL tetracycline for 6 h to induce apoptosis. Whole cell lysates were subjected to SDS-PAGE and immunoblotting analysis using antibodies as indicated. GAPDH serves as the loading control. The asterisk (*) denotes the non-specific band. Experiments were repeated independently for more than three times. **c** Schematic experimental design for the collection and application of cell death conditioned medium. **d** Representative phase-contrast images (left) and quantification of MuSC cell numbers (right) after 48 h culturing. 20,000 freshly isolated MuSCs were seeded in 35 mm dish and cultured with the indicated medium for 48 h. NCM necroptosis conditioned medium, ACM apoptosis conditioned medium. Scale bars, 100 μm. The data are expressed as the means ± SD of 3 independent experiments. **e** Proliferation analysis of NCM-cultured MuSCs by BrdU assay. Freshly isolated MuSCs were cultured in F-10 medium or NCM for 48 h, followed by 10 μM BrdU labeling for 2 h and analyzed by FACS. **f** Quantification of the BrdU^+^ MuSCs as representatively shown in **e**. The data are expressed as the means ± SD of 4 independent experiments. **g** Immunofluorescence staining of Pax7 (green) and MyoD (red) in F-10 or NCM-expanded MuSCs. Cells were cultured and expanded for 2 passages in the corresponding medium followed by immunofluorescence staining as described in Materials and Methods. Nuclei were identified by staining with DAPI. Scale bars, 50 μm. **h** Quantification of the percentages of Pax7^hi^MyoD^low^, Pax7^hi^MyoD^hi^, Pax7^low^MyoD^hi^ and Pax7^low^MyoD^low^ subpopulations of MuSCs as shown in **g**. Histogram represents the percentages of each subpopulation out of 300 cells per condition. Fluorescent intensity was measured by Image Pro-Plus as described in Materials and Methods. The data are expressed as the means ± SD of 6 images. **i** Representative immunofluorescence staining of MyHC (green) in differentiated MuSCs. Nuclei were identified by staining with DAPI. Scale bars, 50 μm. Experiments were repeated independently for more than three times. **j** Representative immunofluorescence staining of Laminin (green) merged with red fluorescent engrafted transplanted MuSCs. Red fluorescent MuSCs isolated from *R26*^*mT/mG*^ transgenic mice were expanded in F-10 medium or NCM for 2 passages and then transplanted into X-ray irradiated recipient, the injured nonfluorescent *Rag2*^−/−^*;Il2rg*^−/−^ TA muscles. Freshly isolated MuSCs were transplanted and used as a positive control. Cross-sections of TA muscles were harvested at 28 days after transplantation and prepared for immunofluorescence staining of Laminin. Nuclei were identified by staining with DAPI. Scale bars, 25 μm. **k** Quantification of the engrafted Tomato^+^ myofibers as shown in **j**. The number of Tomato^+^ myofibers from 24 fields (Leica SP8 microscopy with 20x objective magnification) were quantified for each mouse. Each dot represents an individual mouse. The data are expressed as the means ± SD. *n* = 6 for each group of recipient mice. *P* values for **d** and **k** were determined by unpaired two-tailed *t*-test; *P* value for **f** was determined by unpaired two-tailed *t*-test with Welch’s correction; *P* values for **h** were determined by two-way ANOVA with Tukey’s multiple comparisons test. *P < 0.05; **P < 0.01; ***P < 0.005.

Necroptotic cells are capable of releasing many factors to the extracellular space after membrane breakdown. Then we asked whether the factors released by necroptotic myofibers facilitate MuSC proliferation. To mimic the muscle cell necroptosis in vitro, we generated a tetracycline (Tet) inducible gene expression system to induce *Mlkl* overexpression and necroptosis in C2C12 cell line (Fig. 3a, b), named as C2C12-*Mlkl*-TetON cells. After Tet addition, along with MLKL overexpression (Fig. 3b), necroptosis was efficiently induced. These necroptotic C2C12-*Mlkl*-TetON cells lost membrane integrity as indicated by intranuclear staining of SYTOX Green, and showed greatly reduced viability as measured by intracellular ATP levels (Supplementary information, Fig. S3a, b). After induction of necroptosis, we harvested the necroptosis conditioned medium (NCM) to feed the freshly isolated MuSCs and monitored their proliferation (Fig. 3c). Meanwhile, we also generated Tet-inducible *tBid*-overexpressing C2C12 cell line, named as C2C12-*tBid*-TetON cells, which were capable of undergoing Tet-induced apoptosis (Fig. 3a, b). Apoptosis was efficiently induced in this cell line as indicated by Annexin V staining (Supplementary information, Fig. S3c, d). The apoptosis conditioned medium (ACM) was also harvested to culture the primary MuSCs as a control (Fig. 3c). After 48 h of culturing, we found NCM-cultured MuSCs were able to proliferate and the cell number expanded to nearly 3 fold comparing with the control condition, while ACM did not promote MuSCs proliferation more than the basic medium F-10 did (Fig. 3d). BrdU incorporation assay further confirmed that the population of proliferating MuSCs in NCM-cultured condition was much larger than the basic medium F-10-cultured condition (Fig. 3e, f). These data demonstrate that NCM is capable of promoting MuSC proliferation.

To check whether the NCM-expanded MuSCs maintained the muscle stem cell features, we firstly performed co-immunofluorescence staining of Pax7 with MyoD. Indeed, MuSCs cultured in NCM showed high Pax7 and low MyoD expression levels (Fig. 3g). The percentage of Pax7^hi^ MuSCs cultured in NCM was much higher than those cultured in F-10 medium (Fig. 3h). Moreover, these cells were capable of differentiation in vitro (Fig. 3i). To further test the competency of NCM-cultured MuSCs in vivo, we expanded Tomato-expressing MuSCs in NCM, and then transplanted them into the TA muscles of pre-injured non-fluorescent *Rag2*^−/−^*;Il2rg*^−/−^ recipient mice. Four weeks after transplantation, Tomato^+^ myofibers were detected in the cryo-sections. The NCM-expanded MuSCs displayed substantially higher engraftment efficiency than F-10 medium-cultured MuSCs (Fig. 3j, k). Thus, NCM-expanded MuSCs maintained their stemness both in vitro and in vivo.

### TNC was released by necroptotic myofibers to promote MuSC proliferation

In search of the potential MuSC proliferation-promoting factor(s), we took advantage of our cell-free NCM system for biochemical purification. Before starting the purification, we firstly determined the feature of the factor(s). NCM was pre-incubated with DNase, RNase or trypsin, which were then used to culture the primary MuSCs. Only trypsin treatment inactivated the pro-MuSC-proliferation ability of NCM, while DNase and RNase treatment had no effect on the activity of NCM (Supplementary information, Fig. S4a, b). These results indicate that the MuSC proliferation-promoting factor(s) are most likely to be protein(s) rather than nucleic acids.

**Fig. 4:**
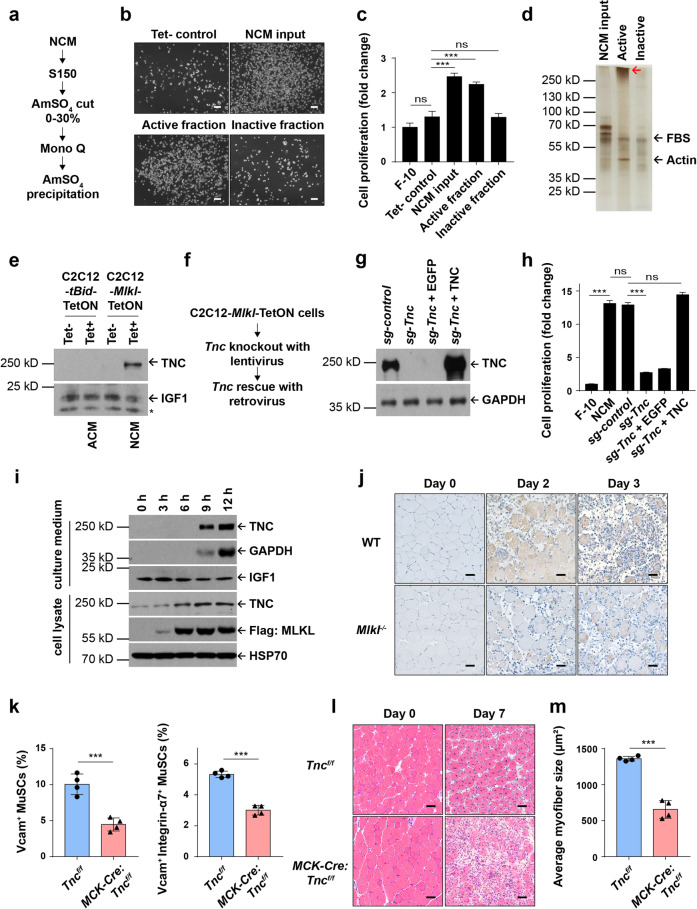
TNC was released by necroptotic myofibers to promote MuSC proliferation. **a** Procedures for purification of the MuSC proliferation-promoting factor(s) from NCM. **b** Representative phase-contrast images of MuSCs cultured in different fractions of NCM after purification. The complete NCM was used as the positive control (NCM input); C2C12-*Mlkl*-TetON medium without tetracycline treatment was used as the negative control (Tet- control). Cells were cultured for 48 h. Scale bars, 100 μm. **c** Quantification of MuSCs that were expanded in different fractions of NCM after purification. Cell proliferation was determined by measuring intracellular ATP levels using CellTiter-Glo assay. The data are expressed as the means ± SD of 3 technical repeats. **d** Identification of TNC as a major effector in the active fraction of NCM. After purification, 8 μg proteins from each fraction were subjected to SDS-PAGE separation, followed by silver staining. The indicated bands were excised and subjected to mass spectrometry analysis. Protein IDs were shown as indicated. The red arrow indicates the position of TNC. **e** Immunoblotting analysis of TNC in ACM and NCM. 10 mL of ACM or NCM were harvested and concentrated using ultra centrifugal filters then subjected to run SDS-PAGE and followed by immunoblotting analysis. IGF1 was used as the loading control. The asterisk (*) denotes the non-specific band. Experiments were repeated independently for more than three times. **f** The knockout-rescue strategy of *Tnc* in C2C12-*Mlkl*-TetON cell line. **g** Immunoblotting analysis of TNC expression in *Tnc* knockout and rescued C2C12-*Mlkl*-TetON cell lines. GAPDH serves as the loading control. **h** Quantification of MuSCs cultured in NCM with or without TNC expressing. NCM was derived from *Tnc* knockout or rescued C2C12-*Mlkl*-TetON cells, as shown in **g**. MuSCs were cultured for 3 passages followed by cell proliferation analysis, which was determined by measuring intracellular ATP levels. The data are expressed as the means ± SD of 3 technical repeats. **i** Immunoblotting analysis of the expression and extracellular release of TNC upon necroptosis induction at different time points as indicated. C2C12-*Mlkl*-TetON cells were treated with 1 μg/mL tetracycline for 12 h to induce necroptosis. Both the whole cell lysates and NCM were harvested and then subjected to immunoblotting analysis using antibodies as indicated. Extracellular release of GAPDH was used as a control to indicate membrane disruption caused by necroptosis; IGF1 was used as the loading control for NCM; HSP70 serves as the loading control for the whole cell lysates. Experiments were repeated independently for more than three times. **j** Representative immunohistochemical staining of TNC in TA muscle cross sections from injured mice (0, 2 and 3 days after CTX injection as indicated). The signals of TNC appear brown in sections counter-stained with hematoxylin (blue). Scale bars, 50 μm. **k** Quantification of the MuSCs in injured TA muscles (3 days after CTX injection) of *Tnc*^*f/f*^ and *MCK-Cre:Tnc*^*f/f*^ mice by FACS analysis. MuSCs were isolated as described in Materials and Methods. Histogram represents the percentage of MuSCs (7-AAD^−^CD11b^−^CD31^−^CD45^−^Sca1^−^Vcam^+^ population, and 7-AAD^−^CD11b^−^CD31^−^CD45^−^Sca1^−^Vcam^+^Integrin-α7^+^ population) out of the total digested mono-nucleus cells. 7-AAD reagent was used to exclude dead cells. *n* = 4 mice for each genotype. The data are expressed as the means ± SD. **l** Representative H&E staining of TA muscle cross-sections from uninjured or injured (7 days after CTX injection) *Tnc*^*f/f*^ and *MCK-Cre:Tnc*^*f/f*^ mice. Scale bars, 50 μm. **m** Quantification of myofiber sizes from CSAs of injured mice 7 days after CTX injection. Histogram graph represents averaged myofiber size. The sizes of each 900 adjacent regenerating myofibers with central nuclei were measured for every mouse. Each dot represents an individual mouse. *n* = 4 mice for each genotype. The data are expressed as the means ± SD. *P* values for **c** and **h** were determined by one-way ANOVA with Tukey’s multiple comparisons test; *P* values for **k** and **m** were determined by unpaired two-tailed *t*-test; ns, non-significant; ***P < 0.005.

We harvested 2 L of NCM from 300 dishes (100 mm) of C2C12-*Mlkl*-TetON cells and subjected it to ammonium sulfate precipitation (Fig. 4a). The 0–30% ammonium sulfate precipitated fraction was able to promote MuSC proliferation (Fig. 4b, c). The crude active fraction was then loaded onto Mono-Q column for anion exchange chromatography analysis. The most effective fractions eluted by 0.1–0.3 M KCl were pooled together for ammonium sulfate precipitation, followed by SDS-polyacrylamide gel electrophoresis (PAGE) analysis and silver staining (Fig. 4d). Three major bands with apparent molecular weight of 250 kD, 60 kD and 40 kD were specifically enriched in the active fraction. These three bands were subjected to mass spectrometry analysis and were identified as TNC, growth supplement components from fetal bovine serum (FBS), and actin. Since FBS components were also detected in the inactive fraction, and actin is ubiquitously expressed in all living cells, we therefore focused on testing the functions of TNC.

The specific presence of TNC in NCM was firstly confirmed by immunoblotting using an antibody against TNC (Fig. 4e). We then characterized the role of TNC in promoting MuSC proliferation. *Tnc* gene was knocked out in C2C12-*Mlkl*-TetON cells with LentiCRISPRv2-mediated CRISPR/Cas9 system (Fig. 4f). The expression of TNC was abolished by sgRNA against *Tnc*, but not control sgRNA, as confirmed by immunoblotting (Fig. 4g). After *Tnc* was knocked out, NCM lost the ability to promote MuSC proliferation, while control sgRNA infection preserved the full capacity of NCM to promote MuSC proliferation (Fig. 4h). When TNC was re-expressed in knockout cells by retrovirus infection (Fig. 4f), the pro-MuSC-proliferation ability of NCM was fully rescued (Fig. 4h). These results demonstrate that TNC is the essential component in NCM to promote MuSC proliferation.

We then checked the spatial-temporal expression pattern of TNC in vitro and in vivo. Both NCM and cell lysate of C2C12-*Mlkl*-TetON cells were collected for immunoblotting analysis. The protein level of TNC increased significantly in the cell lysate along with the overexpression of MLKL upon necroptosis induction (Fig. 4i, lower panel). When necroptotic cells lost their membrane integrity, as indicated by GAPDH release (Fig. 4i, upper panel) or intranuclear staining of SYTOX Green (Supplementary information, Fig. S4c), TNC was released to the NCM (Fig. 4i, upper panel). In the uninjured TA muscles, TNC expression was not detectable (Fig. 4j, Day 0), consistent with the previous reports that TNC was absent in mature myofibers under normal circumstances.^52^ Importantly, both the mRNA and the protein levels of TNC were markedly increased in WT necroptotic myofibers (Fig. 4j and Supplementary information, Fig. S4d, e), whereas the *Mlkl*^−/−^ and *MCK-Cre:Mlkl*^*f/f*^ mice failed to induce TNC expression after injury (Fig. 4j and Supplementary information, Fig. S4f). These results suggest that myofiber necroptosis induces the expression of TNC and its release after membrane rupture.

To further confirm the function of myofiber-derived TNC for muscle regeneration, we generated *MCK-Cre:Tnc*^*f/f*^ mice (Supplementary information, Fig. S4g, h) to specifically knock out *Tnc* gene expression in myofibers and induced injury by CTX injection. As expected, the muscle regeneration defect of *MCK-Cre:Tnc*^*f/f*^ mice resembled the phenotype as we observed in the myofiber necroptosis-deficient mice, specifically in the manner of decreased TNC mRNA transcripts (Supplementary information, Fig. S4i), lack of protein expression (Supplementary Fig. S4j), and decreased MuSCs proliferation on 3 dpi (Fig. 4k). They also depict impaired muscle regeneration as analyzed on 7 dpi (Fig. 4l, m). These data demonstrated that myofiber-derived TNC is required for post-injury MuSC proliferation and muscle regeneration.

### TNC activates EGFR kinase cascade to promote MuSC proliferation

TNC contains an assembly domain, an EGF-like domain, a fibronectin-type III-like domain plus a terminal fibrinogen globe (Supplementary information, Fig. S5a).^53^ We firstly mapped the functional domain of TNC for promoting MuSC proliferation. The truncated forms of TNC (1–700aa, 156–700aa, 700–1980aa, or 1980–2019aa) were re-expressed in the *Tnc* knockout C2C12-*Mlkl*-TetON cells. The expression levels of the truncated TNC were confirmed by immunoblotting (Supplementary information, Fig. S5c). NCM from each cell line was harvested to culture MuSCs, respectively. Only the NCM derived from the N-terminus TNC (1–700aa) expressing cells showed significantly rescued pro-MuSC-proliferation capacity, while NCM derived from the other truncated TNC-expressing cells failed to promote MuSC proliferation (Supplementary information, Fig. S5b). Therefore, the N-terminus region of TNC (1–700aa) conveys the signal of promoting MuSC proliferation.

**Fig. 5:**
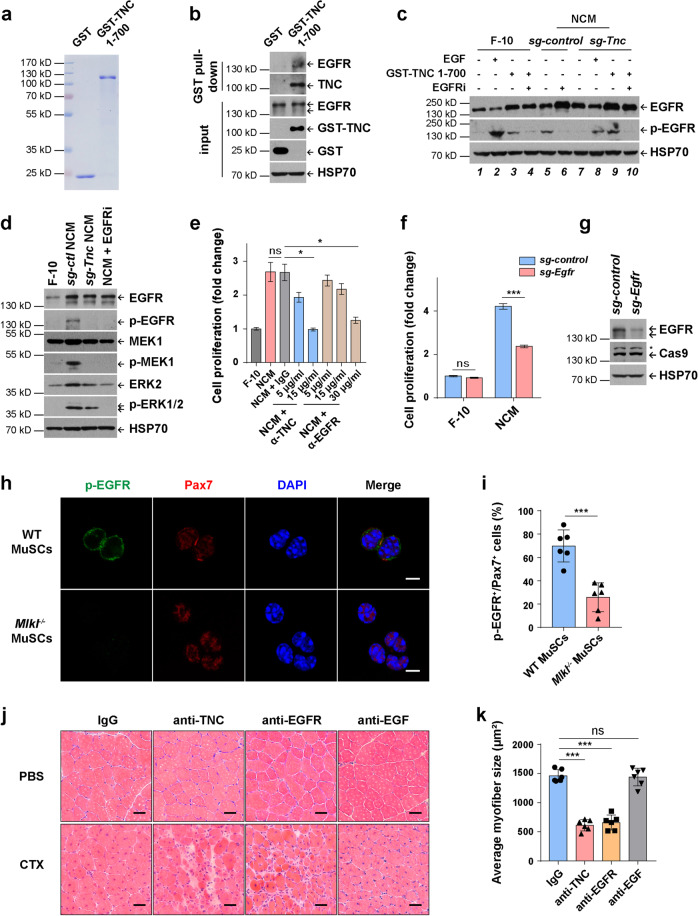
TNC activates EGFR kinase cascade to promote MuSC proliferation. **a** Coomassie blue staining of the purified recombinant mouse TNC (1–700 amino acids). **b** Immunoblotting analysis of the GST pull-down products with the anti-EGFR antibody. 500 ng/mL GST-tagged TNC (1–700aa) was used as the bait to incubate with MuSCs, and subsequently performed GST pull-down assay as described in Materials and Methods. Experiments were repeated independently for more than three times. **c** Immunoblotting analysis of EGFR activation in MuSCs. NCM was derived from both the control (*sg-control*) and *Tnc* knockout (*sg-Tnc*) C2C12-*Mlkl*-TetON cells. MuSCs were treated with combinations of 50 ng/mL EGF, 500 ng/mL GST-TNC-1-700, and 10 μM EGFR inhibitor in F-10 or NCM as indicated for 4 h. Whole cell lysates were subjected to SDS-PAGE and immunoblotting analysis using antibodies as indicated. HSP70 serves as the loading control. Experiments were repeated independently for more than three times. EGFR inhibitor: Afatinib. **d** Immunoblotting analysis of EGFR pathway activation in MuSCs. MuSCs were cultured in different conditions for 48 h. Whole cell lysates were subjected to SDS-PAGE and immunoblotting analysis using antibodies as indicated. F-10, F-10 medium; *sg-ctl* NCM, NCM derived from the control C2C12-*Mlkl*-TetON cells; *sg-Tnc* NCM, NCM derived from the *Tnc* knockout C2C12-*Mlkl*-TetON cells; NCM + EGFRi, normal NCM with EGFR inhibitor (10 μM Afatinib). HSP70 serves as the loading control. Experiments were repeated independently for more than three times. **e** Quantification of MuSCs that were cultured in NCM with TNC or EGFR neutralizing antibodies. MuSCs were cultured for 48 h. Cell proliferation was determined by measuring intracellular ATP levels using CellTiter-Glo assay. The data are expressed as the means ± SD of 3 technical repeats. NCM + α-TNC, NCM with antibody against TNC to neutralize TNC; NCM + α-EGFR, NCM with antibody against EGFR to block EGFR activation. The antibodies were titrated to different doses as indicated. **f** Quantification of *Egfr* knockout MuSCs that were cultured in NCM. MuSCs were isolated from Cas9-expressing mice and infected by AAV encoding sgRNA against *Egfr* (*sg-Egfr)* or the scramble control (*sg-control)*. The *Egfr* and the *control* knockout MuSCs were cultured in F10 or NCM for 48 h. Cell proliferation was determined by measuring intracellular ATP levels using CellTiter-Glo assay. The data are expressed as the means ± SD of 3 technical repeats. **g** Immunoblotting analysis of the knockout efficiency of EGFR in AAV-infected MuSCs. The asterisk (*) denotes the non-specific band. **h** Representative immunofluorescence staining of p-EGFR (green) and Pax7 (red) in freshly isolated MuSCs (2 days after CTX injection). MuSCs were isolated by FACS and fixed on PDL/Collagen-I pre-coated coverslip, as described in Materials and Methods, followed by immunofluorescence staining using the antibodies as indicated. Nuclei were identified by staining with DAPI. Scale bars, 5 μm. **i** Quantification of the p-EGFR^+^/Pax7^+^ cells as shown in **h**. MuSCs isolated from 3 mice were pooled together for immunofluorescence staining in each group. The histogram represents the percentage of p-EGFR^+^/Pax7^+^ cells out of 200 Pax7^+^ cells per genotype. The data are expressed as the means ± SD of 6 images. **j** Representative H&E staining of TA muscle cross sections from injured *Rag2*^−/−^*;Il2rg*^−/−^ mice with antibodies neutralizing TNC/EGFR activation. After CTX injection, individual anti-TNC, anti-EGFR, or anti-EGF antibody was injected intramuscularly every other day as described in Materials and Methods. Twelve days later, TA samples were harvest and prepared for H&E staining. Scale bars, 40 μm. **k** Quantification of myofiber sizes from cross-sectional areas as representative shown in **j**. Histogram graph represents averaged myofiber size. The sizes of each 900 adjacent regenerating myofibers with central nuclei were measured for every mouse. Each dot represents an individual mouse. The data are expressed as the means ± SD. *n* = 6 for each group of mice. *P* values for **e**, **f**, and **k**, were determined by one-way ANOVA with Tukey’s multiple comparisons test; *P* value for **i** was determined by unpaired two-tailed *t*-test with Welch’s correction. ns, non-significant; *P < 0.05; ***P < 0.005.

We purified the recombinant TNC (1–700aa), containing the assembly domain combined with the EGF-like domain (Fig. 5a), and added it into F-10 medium to culture MuSCs. The recombinant TNC (rTNC)-cultured MuSCs were able to proliferate (Supplementary information, Fig. S5d). Moreover, the rTNC-expanded MuSCs maintained their stemness in terms of keeping a higher expression level of Pax7 (Supplementary information, Fig. S5e, f) and normal differentiation ability (Supplementary information, Fig. S5g, h). These cells were also capable of regeneration in vivo after being transplanted into pre-injured *Rag2*^−/−^*;Il2rg*^−/−^ recipient mice (Supplementary information, Fig. S5i, j). Therefore, the N-terminus region of TNC is responsible for activating MuSC proliferation.

One major domain present in the N-terminus of TNC is an EGF-like domain. EGF has been reported to be able to promote cell proliferation of many cell types by activating EGFR signaling pathway.^54^ In skeletal muscle, activation of EGFR signaling pathway can increase the asymmetric division of MuSCs;^55^ EGFR was found to be expressed in MuSCs during activation from quiescence.^56^ We therefore tested whether the EGF-like domain of TNC could serve as an EGF mimic leading to EGF receptor (EGFR) activation in MuSCs. We incubated the purified GST-tagged TNC (1–700aa) with MuSCs, then performed GST pull-down assay. EGFR in MuSCs was pulled down, suggesting the protein-protein interaction between TNC and EGFR (Fig. 5a, b). EGFR signaling in MuSCs was activated as indicated by the presence of phospho-EGFR (p-EGFR) signals; administration of EGFR inhibitor abolished the activation of EGFR signaling induced by recombinant TNC (Fig. 5c). Therefore, administration of recombinant TNC can directly activate EGFR in MuSCs.

The downstream signaling events of EGFR activation — EGFR phosphorylation, MEK phosphorylation, and ERK phosphorylation, were all significantly increased after NCM treatment in MuSCs, while NCM derived from TNC knockout C2C12-*Mlkl*-TetON cells or co-treatment with EGFR inhibitor (EGFRi) could not efficiently activate EGFR signaling (Fig. 5d). We then used monoclonal antibodies against either TNC or EGFR to perform immunoblocking experiments. As shown in Fig. 5e, either TNC or EGFR neutralization disabled NCM to promote MuSC proliferation. Consistently, inhibitors of either EGFR, MEK or ERK blocked the ability of NCM to promote MuSC proliferation (Supplementary information, Fig. S5k). Knocking out EGFR by adeno-associated virus (AAV)-mediated CRISPR/Cas9 in MuSCs also significantly reduced their proliferative capacity in response to NCM (Fig. 5f, g). These results suggest that TNC promoted MuSC proliferation by mimicking EGF to activate EGFR signaling pathway.

Intriguingly, by size exclusion chromatography, we found that the N-terminus assembly domain of TNC drove the EGF-like domain to undergo a radical conformational change, which shifted the monomer into a multimeric presentation. The recombinant EGF-like domain of TNC (156–700 aa) alone was less oligomerized (Supplementary information, Fig. S6a–c), neither activated EGFR in MuSCs (Supplementary information, Fig. S6d) nor promoted their proliferation (Supplementary information, Fig. S6e). These results indicate the polymerization of TNC is crucial for its function in activating EGFR.

To confirm the activation of EGFR signaling pathway by TNC in MuSCs in vivo, we isolated the MuSCs from TA muscles at 2 days after CTX injection, to perform co-immunofluorescence staining of phospho-EGFR with Pax7. Phospho-EGFR signals were detected in WT Pax7^+^ MuSCs but not *Mlkl*^−/−^ Pax7^+^ MuSCs (Fig. 5h, i). Further, we injected either anti-TNC or anti-EGFR antibody intramuscularly in the TA muscles of *Rag2*^−/−^; *Il2rg*^−/−^ mice to block TNC or EGFR function. After CTX-induced injury, TA muscles treated by each of the two antibodies displayed muscle regeneration defects (Fig. 5j, k). In contrast, anti-EGF antibody injection did not affect muscle regeneration (Fig. 5j, k). Consistently, both the mRNA level and the protein level of endogenous EGF was not detected in TA muscles, before or after injury (Supplementary information, Fig. S7). These results suggest that, under pathophysiological conditions, both TNC and EGFR are required for muscle regeneration, while EGF is dispensable.

## Discussion

**Fig. 6:**
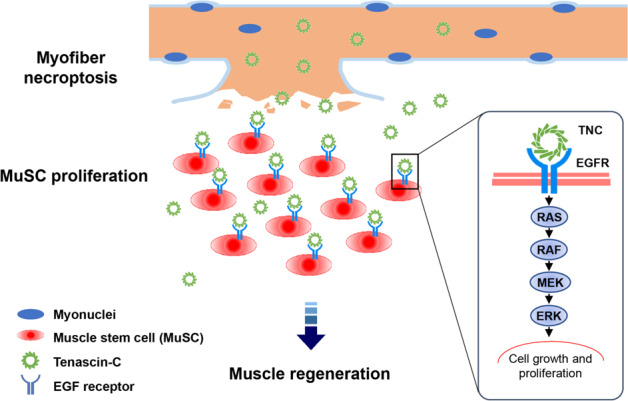
The schematic model of necroptotic myofiber releasing TNC to promote MuSC proliferation. Myofibers commit necroptosis after muscle injury. The necroptotic myofibers express TNC and release it to the MuSC microenvironment following membrane disruption. TNC functions as an EGF mimic through its N-terminus assembly domain combining with EGF-like domain to activate EGFR signaling pathway in MuSCs and promote their proliferation.

Our data revealed a beneficial pathophysiological role of necroptosis in promoting muscle regeneration by releasing TNC to promote MuSC proliferation (as the schematic shown in Fig. 6). The occurrence of necroptosis on one hand clears myofibers in the injury zone, on the other hand actively remodels the components of MuSC microenvironment by releasing factors to facilitate MuSC proliferation. This “one stone, two birds” strategy assures muscle regeneration in a fast responding and economical way. Apoptosis, another form of programmed cell death, has been reported to promote myoblast fusion during the late stage of regeneration.^57^ Therefore, different forms of programmed cell death have distinctive roles during muscle repair.

Across the range of acute injury models (irrespective of the type of acute perturbation, such as CTX or BaCl_2_ injection), we observed myofiber necroptosis happened, and the following regenerative processes were similar. Our finding indicated that examination of necroptosis markers, such as p-MLKL, in samples from patients with muscle trauma will help with diagnostic for muscle regeneration defects. On the other hand, in chronic muscle degenerative disease (Duchenne Muscular Dystrophy, DMD) as previously reported, whether myofibers commit to progressive necroptosis is still controversial.^58,59^ Moreover, a subset of non-competitive MuSCs were found to undergo necroptosis in the *mdx* mice; no MuSC necroptosis was observed in wild type mice after CTX-induced acute injury.^59^ These findings suggested that necroptosis engages in both acute and chronic muscle regeneration, but play different roles depending on the contexts.

Recent literature reported that the transcription upregulation of RIPK3 and MLKL were controlled by SP1, STAT1, or CDK9 in various cancer cell lines^60–62^ and during hepatitis.^63^ JAK/STAT signaling pathway has been shown to play dynamic and important roles during muscle regeneration.^64–67^ It is possible that these transcriptional regulations of necroptosis factors may be shared in necroptotic myofibers after CTX-induced injury. A systematical study of the transcriptional instruction on the expression of necroptosis factors will be one of the most fruitful paths for us to understand the conditional regulation of necroptosis under pathophysiological conditions.

Mechanically, how necroptosis upregulates TNC expression is largely unknown. TNC has a single transcription start site (TSS) localized to the first exon. Sequencing the 5’-flanking region of TNC revealed several potential binding sites for multiple transcription factors.^68^ Reporter activation assay in fibroblasts or cancer cell lines revealed multiple transcription factors regulating TNC gene expression, such as MKL1, OTX2, SP1, JUN, et al.^69–72^ Among all these transcription factors, NF-κB was reported to activate TNC expression in rat cardiac myocytes by mechanical deformation.^73^ It would be interesting to explore how necroptosis triggers the transcription of TNC or other ECM proteins during tissue repair.

Previous studies have shown that acute injury induces infiltration of a series of immune cells to regulate muscle repair.^74–78^ Here we found that necroptosis has a function that is independent of the recruited immune cells upon injury. Furthermore, the production of TNC by necroptotic myofibers and its pro-proliferation functions can be reconstituted in vitro in the absence of immune cells. Combining these findings, necroptosis may affect muscle regeneration from different aspects, either directly through necroptotic myofibers or via attracting non-myofiber cells, such as immune cells.

A striking feature of TNC is its tightly controlled gene expression pattern. It is prominently expressed in embryos, but shows a very restricted distribution pattern in adult tissues, mostly in tendons. The development of skeletal muscle has been reported to be phenotypically normal in *Tnc* knockout mice.^79^ TNC was reported to regulate fetal MuSC regenerative potential in a cell-autonomous manner.^80^ Remarkably, the expression of TNC has been detected in the surrounding areas of several types of adult stem cells: neural stem cells, skin stem cells, and hair follicle stem cells.^52,81^ The temporary expression of TNC has been observed at injury sites of many tissues.^82–84^ TNC is not constitutivly expressed in healthy adult myofibers. Our discovery delineated the mechanism of TNC expression and release that are regulated by necroptotic dying cells at the injury site, and also defined the role of TNC in directly activating MuSC proliferation.

Future studies are warranted to delineate the complexity of the MuSC niche that is provided by necroptotic cell-released factors, including but not necessarily limited to TNC. Moreover, in other tissues, whether a population of terminally differentiated cells also commit MLKL-dependent necroptosis to benefit adjacent stem cell activation for regeneration and homeostasis is worth pursuing in the future.

## Materials and methods

### Animals

Housing, husbandry, and all experimental protocols for mice used in this study were performed in accordance with the guidelines established by the Institutional Animal Care and Use Committee in Shanghai Institute of Biochemistry and Cell Biology (SIBCB), Chinese Academy of Sciences. All animals were housed in individually ventilated cages (IVC) with a temperature (22 ± 1°C) and light (12-h light/dark cycle) controlled specific pathogen-free (SPF) animal facility.

B6.FVB(129S4)-Tg(Ckmm-cre)5Khn/J (*MCK-Cre*) mouse line was purchased from Jackson Laboratory. B6.129(Cg)-*Gt(ROSA)26Sor*^*tm4(ACTB-tdTomato,-EGFP)Luo*^/J (*R26*^*mT/mG*^) mice were gifts from Dr. Yi Arial Zeng in SIBCB. *Rag2*^−/−^; *Il2rg*^−/−^ mice were provided by Dr. Lijian Hui in SIBCB. If not stated specifically, 8–10-week-old male mice were used for all experiments.

### Antibodies

Antibodies used for western blots were purchased from Thermo Fisher Scientific (anti-mtHSP70, cat# MA3–028), Sigma-Aldrich (HRP-anti-FLAG, cat# A8592), Cell Signaling technology (HRP-anti-rabbit IgG, cat# 7074P2; HRP-anti-mouse IgG, cat# 7076; anti-Cleaved Caspase-3, cat# 9661; anti-EGFR, cat# 71655; anti-FLIP, cat# 3210; anti-p-EGFR Y1068, cat# 3777; anti-p-ERK1/2, cat# 9101; anti-p-MEK1/2, cat# 9154; anti-RIP/RIPK1, cat# 3493), Santa Cruz Biotechnology (anti-EGF, cat# sc-374255; anti-ERK2, cat# sc-154; anti-MEK-1, cat# sc-219), Abcam (anti-EGF, cat# ab206423; anti-Tenascin-C, cat# ab3970; anti-Tenascin-C, cat# ab108930; anti-MyoD1, cat# ab64159), ABclonal (anti-GAPDH, cat# AC033; anti-CAS9, cat# A14997; anti-CKM/MCK, cat# A2024; anti-FADD, cat# A5819; anti-IGF1, cat# A11985), GNI (HRP-anti-GST, cat# GNI4310-GT), DSHB (anti-MYH3, cat# F1.652), BD Biosciences (anti-MyoG, cat# 556358), Abgent (anti-MLKL, cat# AP14272b) and ProSci (anti-RIP3/RIPK3, cat# 2283).

Antibodies used for flow cytometry were purchased from Thermo Fisher Scientific (AF700-anti-mouse Sca-1, cat# 56-5981-82), BioLegend (biotin-anti-mouse Vcam, cat# 105704; PE/Cy7-steptavidin, cat# 405206; Pacific Blue-anti-mouse Sca-1, cat#108120), R&D Systems (APC-anti-mouse Integrin a7, cat# FAB3518A), and BD Biosciences (PerCP/Cy5.5-anti-mouse CD11b, cat# 550933; PerCP/Cy5.5-anti-mouse CD31, cat# 562861; PerCP/Cy5.5-anti-mouse CD45, cat# 550944).

Antibodies used for immunofluorescence were purchased from Millipore (MyHC, cat# 05-716; anti-Laminin B2, cat# 05-206), Leica Biosystems (anti-Ki67, cat# NCL_Ki67p), Sigma-Aldrich (anti-laminin, cat# L9393), DSHB (anti-MYH3, cat# F1.652; anti-Pax7, cat# pax7-c), Abgent (anti-MLKL, cat# AP14272b), Abcam (anti-MyoD1, cat# ab64159) and Cell Signaling Technology (anti-p-EGFR Y1068, cat# 3777).

Antibodies used for immunohistochemistry staining were purchased from Abcam (anti-p-MLKL S345, cat# ab196436; anti-Tenascin-C, cat# ab3970).

### Cell culture

293 T and C2C12 cell lines were purchased from and authenticated by ATCC. Cells except MuSCs were cultured at 37 °C with 5% CO_2_ in Dulbecco’s Modified Eagle’s Medium (DMEM. Corning, cat# 10-013-CV) supplemented with 10% fetal bovine serum (FBS. Gibco, cat# 10099141) and 1% penicillin/streptomycin (Gibco, cat# 15140-122). All the cells were verified to be mycoplasma contamination-free using the Mycoplasma Detection Kit (Genloci Biotechnologies, cat# GP5014).

### Whole cell lysate preparation for immunoblotting analysis

Cells were collected, washed with PBS and resuspended in lysis buffer containing 1% TRITON X-100, 150 mM NaCl, 20 mM Tris/HCl (pH 7.4) and 5% glycerol, in the presence of proteinase inhibitor (Roche, cat# 16829800) and phosphatase inhibitor (Thermo Fisher Scientific, cat# A32957). After incubation on ice for 30 min, samples were centrifuged at 16,000× *g* at 4 °C for 10 min. The protein concentration of the supernatant was measured with Bradford Protein Assay Kit (Sangon Biotech, cat# C503031), and a corresponding dose of loading buffer (Thermo Fisher Scientific, cat# NP0007) was added before subjecting to SDS-PAGE. Tissue samples were homogenized in lysis buffer (1% TRITON X-100, 150 mM NaCl, 50 mM Tris/HCl (pH 7.4), 1 mM EDTA-2Na, 1% SDS, 5% glycerol, proteinase inhibitor and phosphatase inhibitor). After centrifugation, the supernatants were boiled with loading buffer. Whole cell lysates of 20–40 μg proteins for each sample were prepared for immunoblotting analysis.

### Muscle injury model

Muscle injury was induced by intramuscular injections of cardiotoxin (CTX, Sigma-Aldrich, cat# C3987) or BaCl_2_ (Sigma-Aldrich, cat# 342920) into TA muscles as previously described.^45,85^ CTX was dissolved in PBS to 10 μM, and BaCl_2_ was dissolved in PBS to 1.2% (w/v). For TA muscle, 3 injections (30 μL for each injection site) were performed for each mouse. The needle (28 gauge) was inserted at a 90’ angle into the skin. Two injection sites were located close to both ends of the TA muscle, and the third injection site was in the middle of the TA muscle. Mice were put under suction anesthesia with isoflurane during surgery.

### In vivo deliveries of cell death inhibitors and antibodies

For in vivo cell death inhibitors delivery, 50 μL of 10 μM necroptosis inhibitor Nec-1s (BioVision, cat# 2263) or 10 μM apoptosis inhibitor z-VAD-fmk (BioVision, cat# 1140) was injected to TA muscles intramuscularly every other day for 7 days, starting from one day before CTX injection. Drugs were diluted in PBS. DMSO was delivered in parallel as the vehicle control.

For in vivo antibody delivery, individual 50 μL of 15 μg/mL monoclonal antibody (anti-EGF, Santa Cruz Biotechnology, cat# sc-374255; anti-Tenascin-C, Abcam, cat# ab3970; anti-EGFR/Cetuximab, MedChemExpress, cat# HY-P9905) was injected into TA muscles of recipient (*Rag2*^−/−^; *Il2rg*^−/−^) mice every other day for 12 days, starting from one day after CTX injection. 50 μL of 15 μg/mL IgG was delivered in parallel as the control.

### Immunofluorescence and immunohistochemistry staining

Frozen section preparation: TA muscle samples were embedded in OCT (Thermo Fisher Scientific, cat# 6506), and frozen in liquid nitrogen. 10 μm thick cryosections were fixed in 4% PFA for 20 min at room temperature and washed twice with PBS, followed by H&E (Haemotoxylin and Eosin) or IF staining.

Immunofluorescence staining: Cryosections were permeabilized in pre-chilled methanol for 6 min at –20 °C. Sections were blocked with M.O.M. Blocking Reagent (Vector, cat# MKB-2213) for 2 h and incubated with primary antibodies at 4 °C for overnight. The next day, the sections were washed three times with PBS and incubated with secondary antibodies for 1 h at room temperature. Then the sections were washed with PBS and counterstained with 0.5 μg/mL DAPI for 1 min and mounted with Antifade Mounting Medium (Vector, cat# H-1000).

Immunohistochemistry staining: freshly isolated TA muscles were fixed in 4% PFA overnight, then dehydrated with a graded ethanol series and xylene, and embedded in paraffin. 5 μm thick sections were deparaffinized and rehydrated. Endogenous hydrogen peroxide was removed with 3% H_2_O_2_, and antigen retrieval was performed in 10 mM citric acid buffer (pH 6.0) with 0.05% TRITON X-100. The sections were then blocked with 2% normal goat serum and incubated with primary antibodies at 4 °C overnight. Samples were then washed with TBST buffer and incubated with HRP-conjugated secondary antibodies for 1 h at room temperature. After washed three times with TBST, the signal was developed with ImmPACT DAB Peroxidase Substrate Kit (Vector, cat# SK-4105). The sections were then counterstained with hematoxylin, dehydrated with ethanol and xylene, and mounted with mounting medium. Images were acquired with the BX53 microscope (Olympus).

### T cell conditioned medium (TCM) collection

T cell conditioned medium (TCM) was generated as previously described.^45^ Briefly, primary splenocytes were harvested from the spleen of 8-week-old mice. After lysis of red blood cells, mono-cells were collected with 40 μm strainers. 4 × 10^7^ splenocytes were cultured in 40 mL RPMI 1640 with 10% FBS, 1% penicillin/streptomycin and 2 μg/mL Concanavalin A (Sigma-Aldrich, cat# C2010) for 72 h. The conditioned medium was filtered through the 0.2 μm filter.

### qRT-PCR

Total RNA was extracted by TRIzol Reagent (Thermo, cat# 15596–018), and reverse transcription was performed using the GoScript Reverse Transcription Mix (Promega, cat# A5790). The quantitative PCR analysis was performed with GoTaq qPCR Master Mix (Promega, cat# A6002) with the CFX96 real-time PCR detection system (Bio-Rad). Results were normalized to the *Gapdh* mRNA level and analyzed by Bio-Rad CFX Manager software. Primers were listed in Supplementary information, Table S2.

### MuSC isolation

Mice were euthanized with CO_2_ and sterilized with 75% ethanol. For each mouse, muscles of the limbs were dissected and digested in 20 mL digestion buffer (DMEM containing 3% penicillin/streptomycin, 1.2 mg/mL Dispase II, Roche, cat# 04942078001, and 2 mg/mL Collagenase Type IV, Thermo Fisher Scientific, cat# 17104019) for 60 min at 37 °C. The digestion was stopped by adding 20 mL of FBS. The digested cells were filtered through 70 μm strainers (BD Falcon, cat# 352350). Red blood cells were lysed by 3 mL RBC lysis buffer (Thermo Fisher Scientific, cat# 00-4333-57) for 2 min and then washed with FACS buffer (PBS containing 1% FBS). Cells were stained by antibody cocktail (PerCP/Cy5.5-CD45/CD11b/CD31, AF700-Sca1, and Biotin-Vcam. 10 μL of each antibody were used) for 45 min on ice, and washed by FACS buffer. Then the cells were re-stained by 10 μL PE/Cy7-streptavidin antibody for another 15 min. Finally, cells were incubated with 0.5 μL of 2 mg/mL propidium iodide (PI. Sigma-Aldrich, cat# 81845) for 5 min, and washed with FACS buffer preparing for FACS analysis. The population of PI^−^PerCP/Cy5.5^−^AF700^−^PE/Cy7^+^ cells were isolated by FACS sorting with Influx (BD Biosciences).

### FACS analysis of MuSCs

All the reagents used here were the same as described in MuSCs isolation. The reaction system was adjusted and specified as follows. TA muscles from 8–10-week-old male mice were dissected and digested with 1 mL muscle digestion buffer for 60 min at 37 °C. The digestion was stopped by adding 2 mL of FBS. The digested cells were filtered through 70 μm strainers. Red blood cells were lysed by 1 mL RBC lysis buffer for 2 min. After staining with antibody cocktails (1 μL each), the mononuclear cells were subjected to FACS analysis using LSRFortessa (BD Biosciences). The population of PI^−^CD45^−^CD11b^−^CD31^−^Sca1^−^Vcam^+^ cells, or 7-AAD^-^CD45^−^CD11b^−^CD31^−^Sca1^−^Vcam^+^Integrin-α7^+^ cells were sorted as MuSCs, detailed in the figure legends. One million cells were used for each analysis.

### Gating strategy for MuSCs

Mono-nuclear cells after digestion as described above, were first gated by FSC/SSC to exclude debris, followed by gating with FSC/Trigger Pulse Width and PI/7-AAD to eliminate non-singlet and dead cells. The population of CD45^−^CD11b^−^CD31^−^Sca1^−^ cells were gated to exclude other cell types. Then Vcam^+^ MuSCs, or Vcam^+^Integrin-α7^+^ MuSCs were gated for ex vivo culturing or quantification as described in the figure legends.

### MuSC expansion and differentiation

Petri dishes were pre-coated with 0.5 mg/mL type I collagen (Corning, cat# 354236, 0.02 M dissolved in acetic acid) overnight, then washed three times with PBS. Ham’s F-10 Nutrient Mixture (Thermo Fisher Scientific, cat# 11550043) with 20% FBS, 1% penicillin/streptomycin, and 10 ng/mL FGF (R and D Systems, cat# 233-FB-025) was used as the base medium. For in vitro expansion, MuSCs were cultured in 50% volume of F-10 base medium combined with 50% volume of conditioned medium (NCM, ACM, or F-10 medium). Cells were passaged every 48 h. For differentiation induction, MuSCs were washed three times with PBS and changed into differentiation medium (DMEM containing 2% horse serum, HyClone, cat# HYCLSH30074.03HI, and 1% penicillin/streptomycin) for 48 h.

### Primary myofiber purification

TA muscles from 8–10-week old male mice were dissected and digested as described in MuSC isolation. After digestion, mononuclear cells were filtered through 40 μm strainers (BD Falcon, cat# 352340), and multi-nuclear cells were harvested as primary fibers.

### Generation of TetON stable cell lines

C2C12-*Mlkl*-TetON cell line was generated by co-infecting C2C12 cells with *pLVX-Tet repressor* and *pLVX-mouse Mlkl S345D/S347D-HA-3xFlag* lentivirus; C2C12-*tBid*-TetON cell line was generated by co-infecting C2C12 cells with *pLVX-Tet repressor* and *pLVX-mouse truncated Bid-HA-3xFlag* lentivirus. Infected cells were selected with 2 μg/mL puromycin (Millipore, cat# 540411) and 500 μg/mL Geneticin (Thermo Fisher Scientific, cat# 1181102). The induced expression of MLKL and tBid was confirmed by immunoblotting analysis with anti-FLAG antibody. The inducible programmed cell death was confirmed by Annexin V/7-AAD FACS analysis (for apoptosis) or SYTOX Green staining (for necroptosis).

### Characterization of apoptosis and necroptosis

For apoptosis analysis, cells were stained with PE-conjugated Annexin V and 7-AAD, according to the manufacturer’s instructions (BD Biosciences, cat# 559763). The stained cells were then analyzed by Celesta flow cytometer (BD Biosciences), and data was processed with FlowJo software. Apoptotic cells (the Annexin V^+^ 7-AAD^-^ population) were quantified based on FACS results.

For necroptosis analysis, the cell impermeable dye SYTOX Green (Thermo Fisher Scientific, cat# S7020, the final concentration of 100 nM) was added into medium 10 min before microscopic analysis. Intranuclear DNA fluorescent staining of SYTOX Green reflects the necroptotic membrane compromise.

### NCM/ACM collection

TetON cell lines were cultured in DMEM with 10% FBS and 1% penicillin/streptomycin. When cell density was around 70%, cells were washed three times with PBS and changed into Opti-MEM (Thermo Fisher Scientific, cat# 31985070) medium with 1 μg/mL tetracycline (MedChemExpress, cat# HY-B0474) and 1% penicillin/streptomycin. For NCM collection, the C2C12-*Mlkl*-TetON culture medium was harvested 12 h later after tetracycline treatment. For ACM collection, the C2C12-*tBid*-TetON culture medium was harvested 6 h after tetracycline treatment. The collected medium was ultra-centrifuged and then filtered by passing through the 0.2 μm filter.

### BrdU/EdU assay

Logarithmically proliferating MuSCs were washed three times with PBS and changed into fresh medium containing 10 μM BrdU (Sigma-Aldrich, cat# B5002). Two hours later, MuSCs were harvested and fixed with ice-cold ethanol at 4 °C overnight. On the second day, cells were permeabilized with 2 M HCl and 0.5% TRITON X-100 for 30 min at room temperature and washed by 0.1 M Na_2_B_4_O_7_ for 5 min. Next, cells were stained with FITC-conjugated anti-BrdU antibody (Thermo Fisher Scientific, cat# 11-5071-42) and PI for 30 min at room temperature, followed by FACS analysis.

In vivo EdU incorporation assay was performed as previously described.^86^ Muscles were injected with CTX, and EdU (100 μg/mouse, dissolved in PBS. RIBOBIO, cat# C00052) was injected intraperitoneally twice (48 h and 24 h before sacrifice). After digestion, MuSCs were isolated as the 7-AAD^−^CD45^−^CD11b^−^CD31^−^Sca1^−^Vcam^+^Integrin-α7^+^ population by FACS. MuSCs were then fixed, and EdU was stained with Cell-Light Apollo 567 Stain Kit according to the manufacturer’s instructions (RIBOBIO, cat# C10371-1). The percentage of EdU^+^ MuSCs was analyzed in BD LSRFORTESSA.

### Protein purification from NCM and mass spectrometry

A total volume of 2 L necroptosis conditioned medium (derived from 300 plates of 10 cm dish) was harvested. Insoluble substances were removed by 150,000× *g* centrifugation. The medium was fractionated with ammonium sulfate according to the guidance, as previously reported.^87^ Precipitates of each fraction were resuspended and dialyzed with pH 8.0 buffer containing 100 mM KCl and 25 mM HEPES for overnight. The active protein samples (precipitated by 0–30% ammonium sulfate) were loaded onto Mono-Q column (GE Healthcare, cat# 17-5166-01) for anion exchange chromatography analysis. The binding proteins were eluted with KCl gradient buffer (0.1–1 M) containing 25 mM HEPES (pH 8.0). Eluted proteins were re-precipitate with ammonium sulfate and subjected to run SDS-PAGE followed by silver staining. Designated bands from the gel were cut into pieces and prepared for mass spectrometry analysis. All operations were performed in 4 °C cold room.

After each step of purification, samples were filtered through 0.2 μm filter. The bio-activity of each fraction was measured by culturing MuSCs to promote their proliferation.

Detailed protein hit IDs were listed in Supplementary information, Table S1.

### Cell survival assay and growth curve calculation

For MuSCs, 1000 cells in 100 μL medium were seeded into each well of 96-well plate. For C2C12-TetON cells, 3000 cells were seeded into each well of the 96-well plate and grew for 24 h. Tetracycline was added to reach a final concentration at 1 μg/ml. 6 h (for apoptosis induction) or 12 h (for necroptosis induction) later, cell survival was determined by measuring the intracellular ATP level of total cells measured with CellTiter-Glo Luminescent Cell Viability Assay Kit (Promega, cat# 7573) according to the manufacturer’s instructions.

To monitor the growth curve of MuSCs, 20,000 primary MuSCs were seeded into each well of 6-well plate. Every 48 h, cells were digested with trypsin and counted, and then 20,000 cells were passaged. The total cell number was determined by multiplying each passage cell number with the expanding fold.

### Virus-mediated gene knockout

sgRNA sequences (designed on http://crispor.tefor.net/) were cloned into LentiCRISPRv2. Lentivirus was packaged in 293 T cells. C2C12 cells were incubated with the concentrated virus in the presence of 1 μg/mL hexadimethrine bromide (Sigma, cat# H9268). Cell selection was initiated by using 200 μg/mL hygromycin B (Selleck, cat# S2908) for at least one week. Knockout efficiency was confirmed by qPCR or immunoblotting analysis.

For *Egfr* knockout in MuSCs, Cas9 expressing MuSCs were isolated from *PB-A-Cas9* mice and expanded in T cell conditioned medium. Then cells were infected with adeno-associated virus (AAV) carrying Tomato and *sg-control* or *sg-Egfr*. Tomato^+^ MuSCs were sorted by FACS.

Primers of sgRNA were listed in Supplementary information, Table S3.

### MuSC transplantation

MuSCs from *R26*^*mT/mG*^ transgenic mice were sorted by FACS and expanded in vitro. The recipient *Rag2*^−/−^*;Il2rg*^−/−^ mouse legs were irradiated with 18 G X-ray to eliminate endogenous MuSCs. On the day after irradiation, TA muscles of recipient mice were injected with CTX, followed by 50,000 MuSCs transplantation (suspended in 50 μL PBS). Mice were allowed to get recovered for 28 days. The TA muscle cross sections were harvested and prepared for immunofluorescence staining with anti-Laminin alpha-2 antibody. The Tomato^+^ myofibers were counted under Leica SP8 microscopy as successfully engrafted cells.

### Recombinant TNC purification

Plasmids (*pGEX-6P-1-mouse Tnc 2-700* or *pGEX-6P-1-mouse Tnc 156-700*) were transformed into BL21 (DE3) competent E. coli cells. 1 L cultures of BL21 each containing a different protein expression constructs were grown at 37 °C in LB and induced with 1 mM IPTG (AMRESCO, cat# 0487) at OD_600_ = 0.4. Cells were incubated at 16 °C overnight and harvested. Cells were lysed in 80 mL lysis buffer (150 mM NaCl, 50 mM Tris/HCl pH 8.0, 1% glycerol, 200 μg/mL PMSF and 0.2 mM β-mercaptoethanol) and centrifuged. The cleared lysates were incubated with 4 mL glutathione-coupled Sepharose 4B beads (GE Healthcare, cat# 17-0756-01) at 4 °C overnight, and the beads were subsequently washed for three times then eluted with 30 mM GSH. The eluted proteins were loaded onto a Superose 6 Increase 10/300 GL (GE Healthcare, cat#29-0915-96) equilibrated in PBS/Az using an ÄKTA FPLC system at 0.3 mL/min and the A_280_ of the eluate were monitored continuously. Mass standards used here were from a commercial HMW calibration kit (GE Healthcare).

### GST-pull down assay

GST or GST-tagged recombinant TNC (500 ng/mL) was added into the culture medium and incubated with MuSCs. 4 h later, cells were washed and harvested. Whole cell lysates were prepared as described above. 15 μL glutathione-coupled Sepharose 4B beads were added and incubated with the whole cell lysates at 4 °C for overnight. Then beads were washed three times with lysis buffer and boiled in 100 μL 1× loading buffer and subjected to run SDS-PAGE.

### Statistical analyses

The number of biological replicates and technical repeats in each experimental group is indicated in figure legends. The experimental design incorporated user blinding when possible. Compiled data are expressed as means ± standard deviation (SD). For comparison between two groups, a two-tailed unpaired Student’s *t*-test was used when variances were similar (tested with F-test); whereas a two-tailed unpaired Student’s *t*-test with Welch’s correction was used when variances were different; for multiple comparisons, a one-way ANOVA test was used, followed by Dunnett’s post-test when comparing each group to the control group, or followed by Tukey’s post-test when comparing all pairs of groups. Statistical analysis was performed in GraphPad Prism 7 (GraphPad Software) or Microsoft Excel (Microsoft).

## Supplementary information

Supplementary information, Fig. S1

Supplementary information, Fig. S2

Supplementary information, Fig. S3

Supplementary information, Fig. S4

Supplementary information, Fig. S5

Supplementary information, Fig. S6

Supplementary information, Fig. S7

Supplementary information, Table S1

Supplementary information, Table S2

Supplementary information, Table S3
