# Supplementary material for: Myofiber necroptosis promotes muscle stem cell proliferation via releasing Tenascin-C during regeneration
Source: Cell Res. 2020 Aug 24;30(12):1063–77. doi: 10.1038/s41422-020-00393-6 (PMC7784988; doi:10.1038/s41422-020-00393-6)
Supplement: Supplementary file 1 — Supplementary information, Fig. S1 [file 41422_2020_393_MOESM1_ESM.pdf]

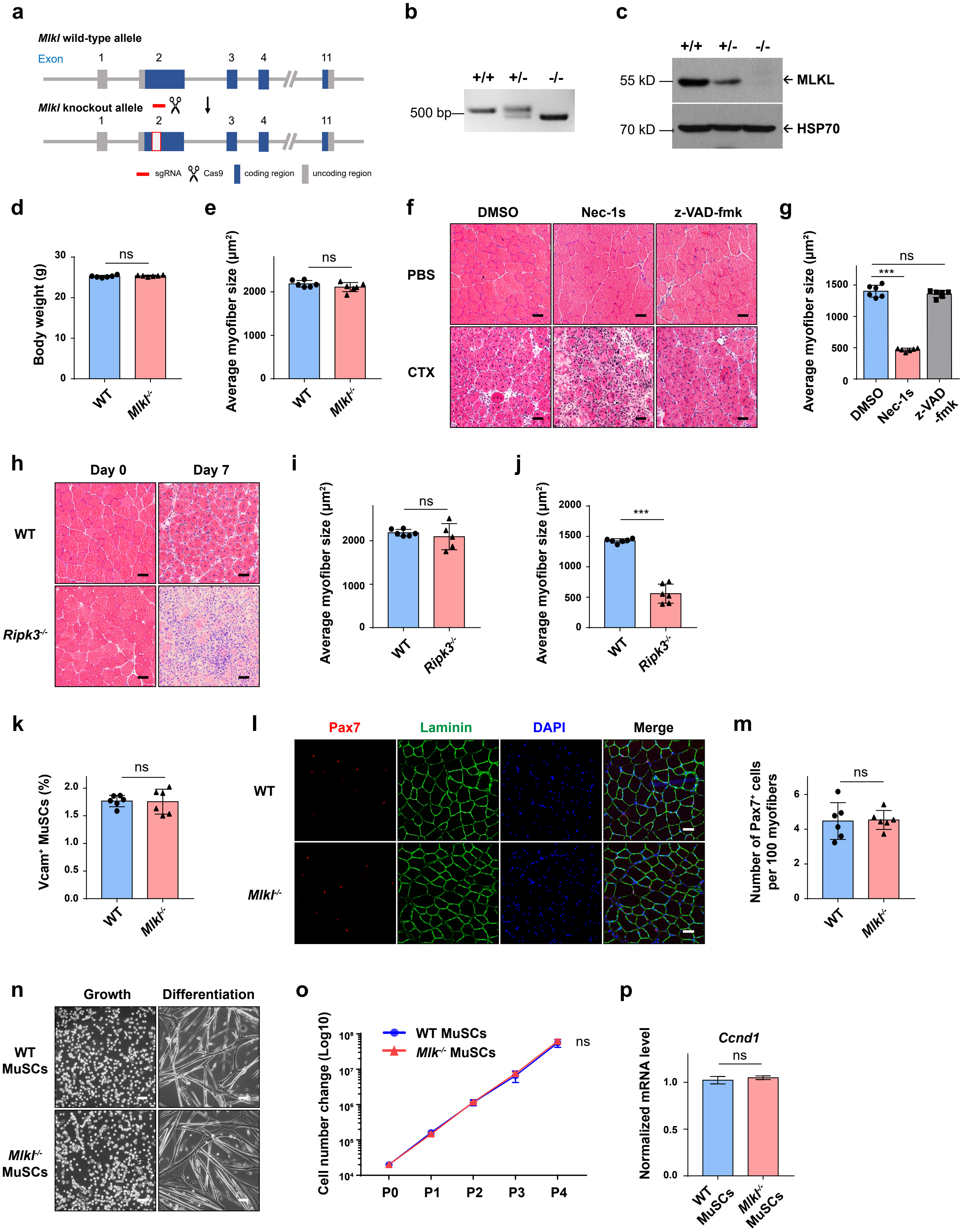

Zhou *et al.*, Figure S1

Supplementary information, Fig S1. | Necroptosis deficiency does not affect early muscle development.

a Schematic strategy of generating *Mlkl*-deficient mice using CRISPR/Cas9 system.

b Genotyping of *Mlkl*$^{-/-}$ mice and their littermates. The shorter PCR product indicates an 87-bp deletion in exon 2 of the *Mlkl* gene.

c Immunoblotting analysis of MLKL expression in mouse dermal fibroblasts (MDF) generated from *Mlkl*$^{-/-}$ mice and their littermates.

d Body weights of WT and *Mlkl*$^{-/-}$ mice at 8 weeks of age. The data are expressed as the mean ± SD. n = 6 each for WT and *Mlkl*$^{-/-}$ mice.

e Quantification of myofiber sizes from cross-sectional areas (CSAs) of uninjured mice. Histogram graph represents averaged myofiber size. The sizes of each 900 adjacent myofibers were measured for every mouse. Each dot represents an individual mouse. The data are expressed as the mean ± SD. n = 6 each for WT and *Mlkl*$^{-/-}$ mice.

f Representative Hematoxylin and Eosin (H&E) staining of TA muscle cross-sections from injured (7 days after CTX injection) mice treated with different cell death inhibitors. Cell death inhibitors were injected intramuscularly every other day, starting from one day before PBS/CTX injection. DMSO, the vehicle dimethyl sulfoxide; Nec-1s, the necroptosis inhibitor Necrostatin-1 stable variant; z-VAD-fmk, the apoptosis inhibitor. Scale bars: 50 µm.

g Quantification of myofiber sizes from TA muscle cross sections as representatively shown in **f**. The sizes of each 900 adjacent regenerating myofibers with central nuclei were measured for every mouse. Each dot represents an individual mouse. The data are expressed as the mean ± SD. n = 6 for each group of mice.

h Representative H&E staining of TA muscle cross sections from both uninjured (Day 0) and injured (7 days after CTX injection) mice. Scale bar: 50 µm.

i Quantification of myofiber sizes from cross-sectional areas (CSAs) of uninjured mice. Histogram graph represents averaged myofiber size. The sizes of each 900 adjacent myofibers were measured for every mouse. Each dot represents an individual mouse. The data are expressed as the mean ± SD. n = 6 for WT mice; n = 5 for *Ripk3*$^{-/-}$ mice.

j Quantification of myofiber sizes from cross-sectional areas (CSAs) of injured mice (7 days after CTX injection, as representatively shown in **h**). The sizes of each 900 adjacent regenerating myofibers with central nuclei were measured for every mouse. Each dot represents an individual mouse. The data are expressed as the mean ± SD. *n* = 6 each for WT and *Ripk3*$^{-/-}$ mice.

k Quantification of the MuSCs from uninjured TA muscles by FACS analysis. MuSCs were isolated as described in Methods. Histogram represents the percentage of MuSCs (PI^{-}CD11b^{-}CD31^{-}CD45^{-}Sca1^{-} Vcam^{+} population) out of the total digested mono-nucleus cells. Each dot represents an individual mouse. The data are expressed as the mean ± SD. *n* = 6 each for WT and *Mlkl*$^{-/-}$ mice.

l Representative immunofluorescence staining of Pax7 (red) and Laminin (green) in TA muscle cross sections from uninjured mice. Nuclei were identified by staining with DAPI. Scale bars: 50 μm.

m Quantification of the Pax7^{+} MuSCs as shown in **l**. The histogram represents the number of Pax7^{+} cells out of 100 myofibers per genotype. The data are expressed as the mean ± SD of 3 images. *n* = 6 each for WT and *Mlkl*$^{-/-}$ mice.

n Representative phase-contrast images of *in vitro* expanded or differentiated MuSCs. MuSCs were isolated from WT and *Mlkl*$^{-/-}$ mice, as described in the Methods. MuSCs isolated from 3 mice were pooled together for *in vitro* expansion and differentiation. Freshly isolated MuSCs were cultured in T cell conditioned medium for expansion or differentiation medium for differentiation, respectively. Scale bars: 100 μm.

o Growth curve of MuSCs expanded in T cell conditioned medium. Cells were passaged every other day. The data are expressed as the mean ± SD of 3 independent experiments.

p qRT-PCR analysis of *Ccnd1* mRNA level of P4 MuSCs as shown in **o**. The mRNA level of *Gapdh* was used as the internal control. The data are expressed as the mean ± SD of 3 technical repeats.

P values for **d**, **e**, **g**, **i**, **j**, **k**, **m,** and **p** were determined by unpaired two-tailed *t*-test; *P* values for **o** were determined by two-way ANOVA. ns, non-significant; *** $P < 0.005$.
