# Supplementary material for: Myofiber necroptosis promotes muscle stem cell proliferation via releasing Tenascin-C during regeneration
Source: Cell Res. 2020 Aug 24;30(12):1063–77. doi: 10.1038/s41422-020-00393-6 (PMC7784988; doi:10.1038/s41422-020-00393-6)
Supplement: Supplementary file 2 — Supplementary information, Fig. S2 [file 41422_2020_393_MOESM2_ESM.pdf]

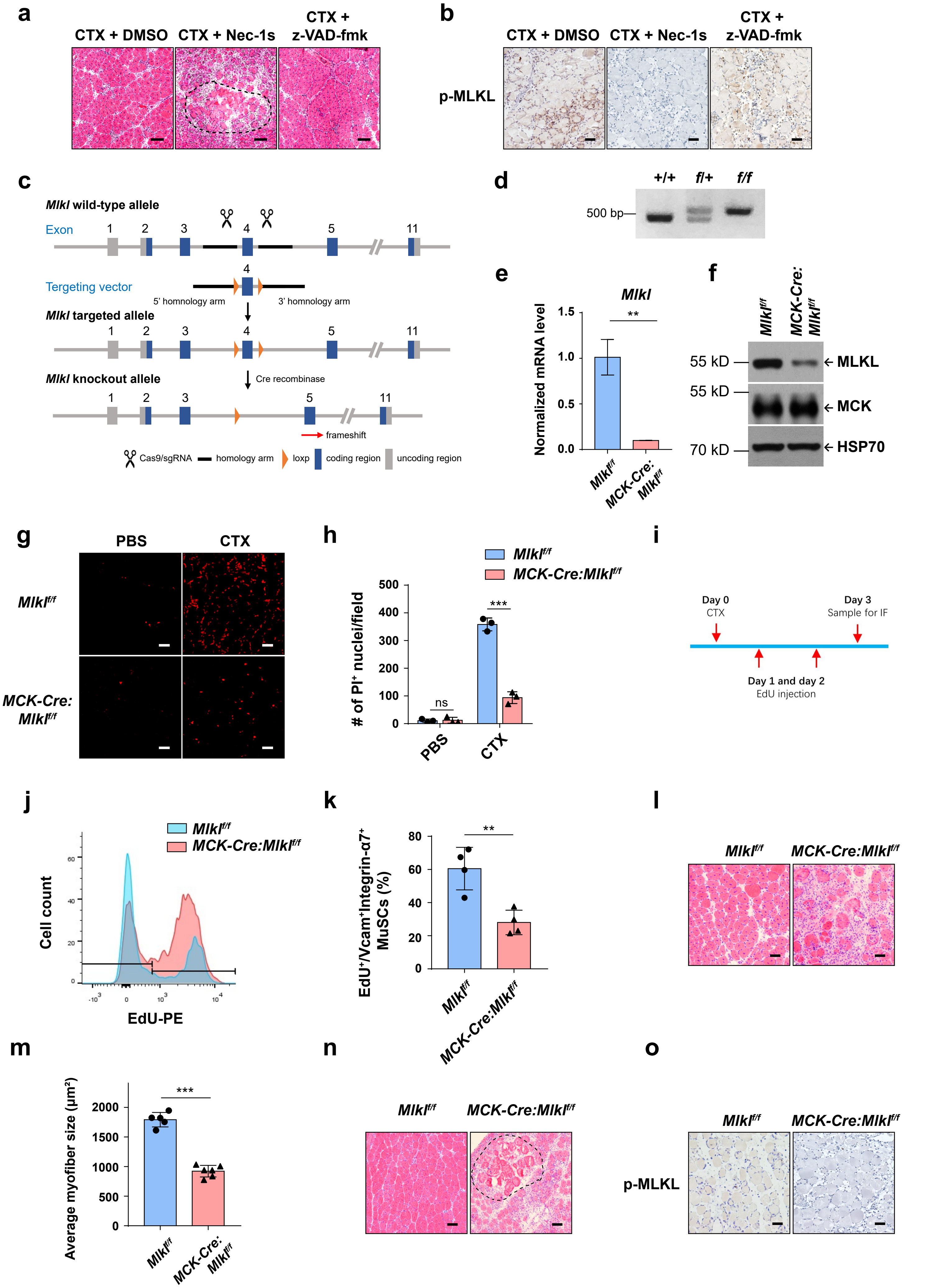


Zhou *et al.*, Figure S2

**Supplementary information, Fig S2. | Myofibers are committed necroptosis after muscle injury.**

**a** Representative H&E staining of TA muscle cross-sections from injured mice (7 days after CTX injection) treated with different cell death inhibitors. Cell death inhibitors were injected intramuscularly every other day, starting from one day before CTX injection. The continuous dotted line delineates the borders of death-resistant myofibers. DMSO, the vehicle dimethyl sulfoxide; Nec-1s, the necroptosis inhibitor Necrostatin-1 stable variant; z-VAD-fmk, the apoptosis inhibitor. Scale bars: 100 μm.

**b** Representative immunohistochemical staining of p-MLKL in TA muscle cross sections from injured mice (2 days after CTX injection) treated with cell death inhibitors. Cell death inhibitors were injected intramuscularly twice: one day before CTX injection, and one day after CTX injection. The signals of p-MLKL appear brown in sections counter-stained with hematoxylin (blue). DMSO, the vehicle dimethyl sulfoxide; Nec-1s, the necroptosis inhibitor Necrostatin-1 stable variant; z-VAD-fmk, the apoptosis inhibitor. Scale bars: 50 μm. Experiments were repeated independently for more than three times.

**c** Schematic strategy of generating *Mlkl*$^{f/f}$ mice using the CRISPR/Cas9 system.

**d** Genotyping of *Mlkl*$^{f/f}$ mice and their littermates. The longer PCR products indicate the *Flox* insertion in exon 4 of the *Mlkl* gene.

**e** qRT-PCR analysis of *Mlkl* mRNA level in injured TA muscles (3 days after CTX injection) from *Mlkl*$^{f/f}$ and *MCK-Cre;Mlkl*$^{f/f}$ mice. The mRNA level of *Gapdh* was used as the internal control. TA samples from 3 mice were pooled together for qRT-PCR analysis. The data are expressed as the mean ± SD of 3 technical repeats.

**f** Immunoblotting analysis of MLKL and MCK expression in TA muscle samples from *Mlkl*$^{f/f}$ and *MCK-Cre;Mlkl*$^{f/f}$ mice at 3 days after CTX injection. TA lysates extracted from 3 mice were pooled together for each group. HSP70 serves as the loading control. Experiments were repeated independently for more than three times.

**g** Immunofluorescence staining of PI (Propidium Iodide) in TA muscle cross-sections of *Mlkl*$^{f/f}$ and *MCK-Cre;Mlkl*$^{f/f}$ mice 1 day after CTX injection. PI (dissolved in PBS, 50 μg/per mice) was injected intravenously at 30 minutes before sacrifice. Scale bars: 50 μm.

**h** Statistical analysis of $PI^+$ nuclei per field. The number of $PI^+$ nuclei from 6 fields (Leica SP8 microscopy with 20x objective magnification per field) were quantified for each mouse. Each dot represents an individual mouse. The data are expressed as the mean ± SD. *n* = 3 mice for each group.

**i** Schematic strategy of *in vivo* EdU incorporation experiment. TA muscles of *Mlkl*$^{f/f}$ and *MCK-Cre:Mlkl*$^{f/f}$ mice were injected with 100 µL of 10 µM CTX at day 0. On day 1 and 2 post CTX injection, 100 µg of EdU (dissolved in 200 µL PBS) was injected intraperitoneally. On day 3 post CTX injection, mice were sacrificed, and MuSCs were isolated (7-AAD$^-$CD11b$^-$CD31$^-$CD45$^-$Sca-1$^-$Vcam$^+$Integrin-α7$^+$ population) by FACS and subjected for EdU staining.

**j** Representative FACS data of EdU$^+$ MuSCs out of the Vcam$^+$Integrin-α7$^+$ population from *Mlkl*$^{f/f}$ and *MCK-Cre:Mlkl*$^{f/f}$ mice 3 days after CTX injection. Vcam$^+$Integrin-α7$^+$ MuSCs were isolated by FACS (7-AAD$^-$CD11b$^-$CD31$^-$CD45$^-$Sca-1$^-$Vcam$^+$Integrin-α7$^+$ population), fixed with 4% PFA, stained with the EdU staining kit, and analyzed by FACS.

**k** Quantification of EdU$^+$/Vcam$^+$Integrin-α7$^+$ MuSCs as shown in **j**. MuSCs isolated from 3 mice were pooled together for FACS analysis. The data are expressed as the mean ± SD of 3 technical repeats.

**l** Representative H&E staining of TA muscle cross-sections from *Mlkl*$^{f/f}$ and *MCK-Cre;Mlkl*$^{f/f}$ mice 8 days after $BaCl_2$ injection. 100 µL of 1.2% $BaCl_2$ dissolved in PBS was injected per TA. Scale bars: 50 µm.

**m** Quantification of myofiber sizes from cross-sectional areas (CSAs) of injured mice (8 days after $BaCl_2$ injection, as representatively shown in **i**). The sizes of each 900 adjacent regenerating myofibers with central nuclei were measured for each mouse. *n* = 5 for *Mlkl*$^{f/f}$ mice and *n* = 6 for *MCK-Cre:Mlkl*$^{f/f}$ mice. The data are expressed as the mean ± SD.

**n** Representative H&E staining of TA muscle cross-sections from *Mlkl*$^{f/f}$ and *MCK-Cre:Mlkl*$^{f/f}$ mice 8 days after $BaCl_2$ injection. The continuous dotted line delineates the borders of cell death-resistant myofibers. Scale bars: 100 µm.

**o** Representative immunohistochemical staining of p-MLKL in TA muscle cross sections from *Mlkl*$^{f/f}$ and *MCK-Cre:Mlkl*$^{f/f}$ mice 2 days after $BaCl_2$ injection. The signals of p-MLKL appear brown in sections counter-stained with hematoxylin (blue). Scale bars: 50 µm.

*P* values for **e** and **k** were determined by unpaired two-tailed *t*-test; *P* values for **h** were determined by one-way ANOVA with Tukey's multiple comparisons test; *P* value for **m** was determined by unpaired two-tailed *t*-test with Welch's correction. ns, non-significant; ** $P < 0.01$; *** $P < 0.005$.
