# Supplementary material for: Myofiber necroptosis promotes muscle stem cell proliferation via releasing Tenascin-C during regeneration
Source: Cell Res. 2020 Aug 24;30(12):1063–77. doi: 10.1038/s41422-020-00393-6 (PMC7784988; doi:10.1038/s41422-020-00393-6)
Supplement: Supplementary file 3 — Supplementary information, Fig. S3 [file 41422_2020_393_MOESM3_ESM.pdf]

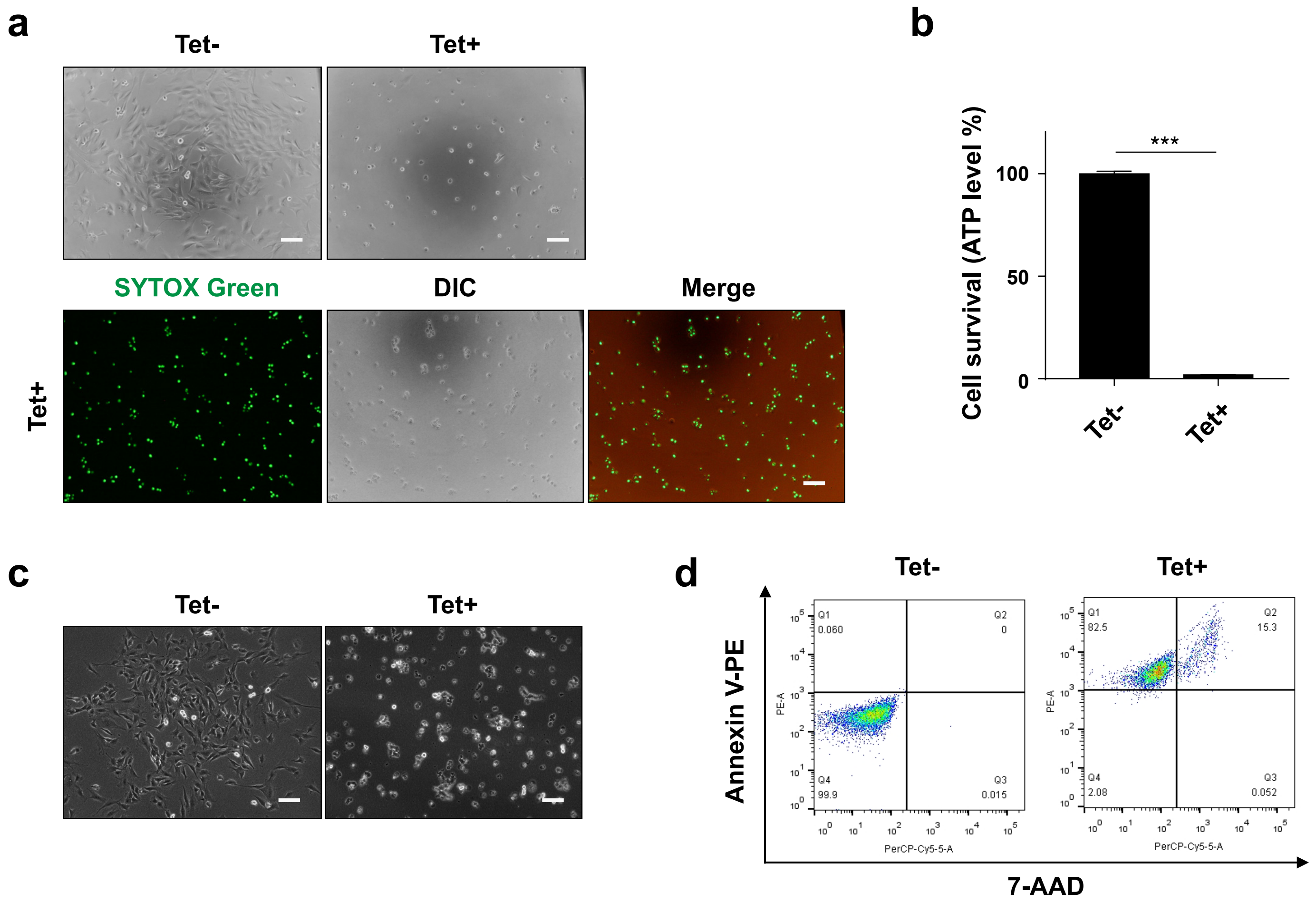

Zhou *et al.*, Figure S3

Supplementary information, Fig S3. | Characterization of C2C12 cell lines that are capable of being induced to undergo apoptosis or necroptosis.

a Representative bright-field (upper) and fluorescent (lower) images of C2C12-*Mlkl*-TetON cells treated with 1 μg/mL tetracycline for 12 hours to induce necroptosis. The cell impermeable dye SYTOX Green was used to indicate membrane disruption during necroptosis. Scale bars: 100 μm.

b Cell survival analysis of necroptotic C2C12-*Mlkl*-TetON cells, as shown in **a**. Cell survival was determined by measuring intracellular ATP levels by CellTiter-Glo assay. The data are expressed as the mean ± SD of 3 technical repeats. *P* values were determined by unpaired two-tailed *t*-test with Welch's correction. *** $P < 0.005$.

c Representative phase-contrast images of C2C12-*tBid*-TetON cells treated with 1 μg/mL tetracycline for 6 hours to induce apoptosis. Scale bars: 100 μm.

d FACS analysis of apoptotic (Annexin V^-/7-AAD^+ population) C2C12-*tBid*-TetON cells as shown in **c**.
