# Supplementary material for: Myofiber necroptosis promotes muscle stem cell proliferation via releasing Tenascin-C during regeneration
Source: Cell Res. 2020 Aug 24;30(12):1063–77. doi: 10.1038/s41422-020-00393-6 (PMC7784988; doi:10.1038/s41422-020-00393-6)
Supplement: Supplementary file 4 — Supplementary information, Fig. S4 [file 41422_2020_393_MOESM4_ESM.pdf]

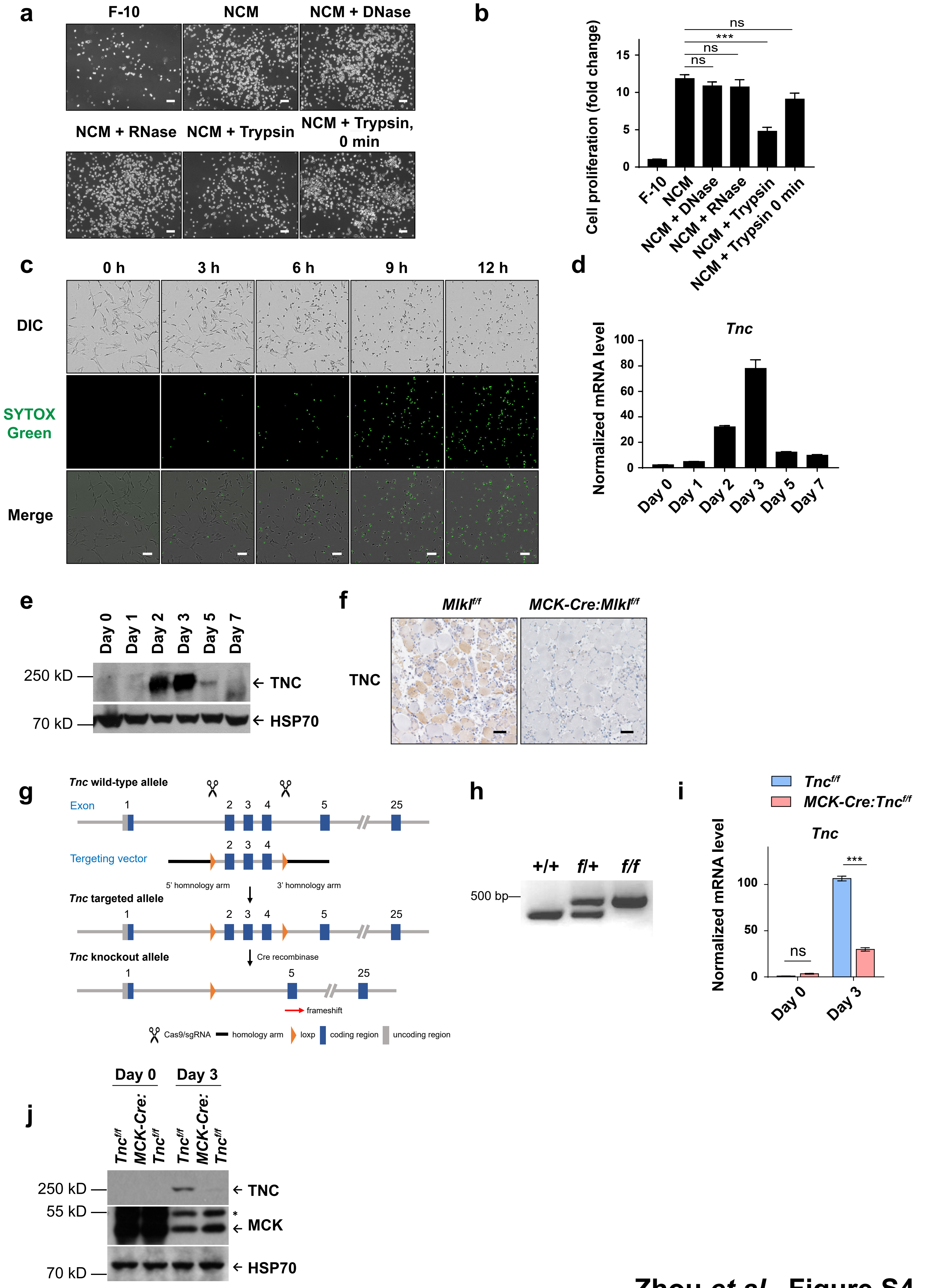


Zhou *et al.*, Figure S4

**Supplementary information, Fig S4. | TNC is upregulated during necroptosis and released to the extracellular space after membrane disruption.**

**a** Representative phase-contrast images of MuSCs cultured in NCM containing different enzymes. Cells were cultured for 3 passages. Scale bars: 100 μm.

**b** Quantification of MuSCs that were expanded in different conditions as shown in **a**. Cell proliferation was determined by measuring intracellular ATP levels by CellTiter-Glo assay. All ATP levels were normalized with F-10 medium cultured condition. The data are expressed as the mean ± SD of 3 technical repeats.

**c** Live-cell imaging of C2C12-*Mlkl*-TetON cells undergoing necroptosis after 1 μg/mL tetracycline treatment. The cell impermeable dye SYTOX Green was used to indicate membrane disruption during necroptosis. Scale bars: 100 μm.

**d** qRT-PCR analysis of *Tnc* mRNA level in injured TA muscle at the indicated days after CTX injection. The mRNA level of *Gapdh* was used as the internal control. TA muscle samples extracted from 3 mice were pooled together in each group for qRT-PCR analysis. The data are expressed as the mean ± SD of 3 technical repeats.

**e** Immunoblotting analysis of TNC expression in myofibers at different time points after muscle injury. Myofibers were purified from TA muscles as described in the Methods. Myofibers were purified from TA muscles as described in the Methods. Whole myofiber lysates were exacted from 3 mice and pooled together for each condition. HSP70 serves as the loading control. Experiments were repeated independently for more than three times.

**f** Representative immunohistochemical staining of TNC in TA muscle cross sections from injured (2 days after CTX injection) *Mlkl*$^{f/f}$ and *MCK-Cre:Mlkl*$^{f/f}$ mice. The signals of TNC appear brown in sections counter-stained with hematoxylin (blue). Scale bars: 50 μm.

**g** Schematic strategy of generating *Tnc*$^{f/f}$ mice using the CRISPR/Cas9 system.

**h** Genotyping of *Tnc*$^{f/f}$ mice and their littermates. The longer PCR product indicates loxp insertion out of exons 2-4 of the *Tnc* gene.

**i** qRT-PCR analysis of Tnc mRNA level in uninjured TA muscles and TA muscles 3 days after CTX injection from *Tnc*$^{f/f}$ and *MCK-Cre:Tnc*$^{f/f}$ mice. The mRNA level of *Gapdh* was used as the internal

control. TA samples from 2 mice were pooled together for qRT-PCR analysis. The data are expressed as the mean ± SD of 3 technical repeats.

**j** Immunoblotting analysis of TNC and MCK expression in uninjured TA muscles and TA muscles 3 days after CTX injection. Whole TA muscle lysates were exacted from 2 mice and pooled together for each condition. HSP70 serves as the loading control. The asterisk (*) denotes the non-specific band.

*P* values for **b** and **i** were determined by one-way ANOVA with Tukey's multiple comparisons test. ns, non-significant; *** $P < 0.005$.
