# Supplementary material for: Myofiber necroptosis promotes muscle stem cell proliferation via releasing Tenascin-C during regeneration
Source: Cell Res. 2020 Aug 24;30(12):1063–77. doi: 10.1038/s41422-020-00393-6 (PMC7784988; doi:10.1038/s41422-020-00393-6)
Supplement: Supplementary file 5 — Supplementary information, Fig. S5 [file 41422_2020_393_MOESM5_ESM.pdf]

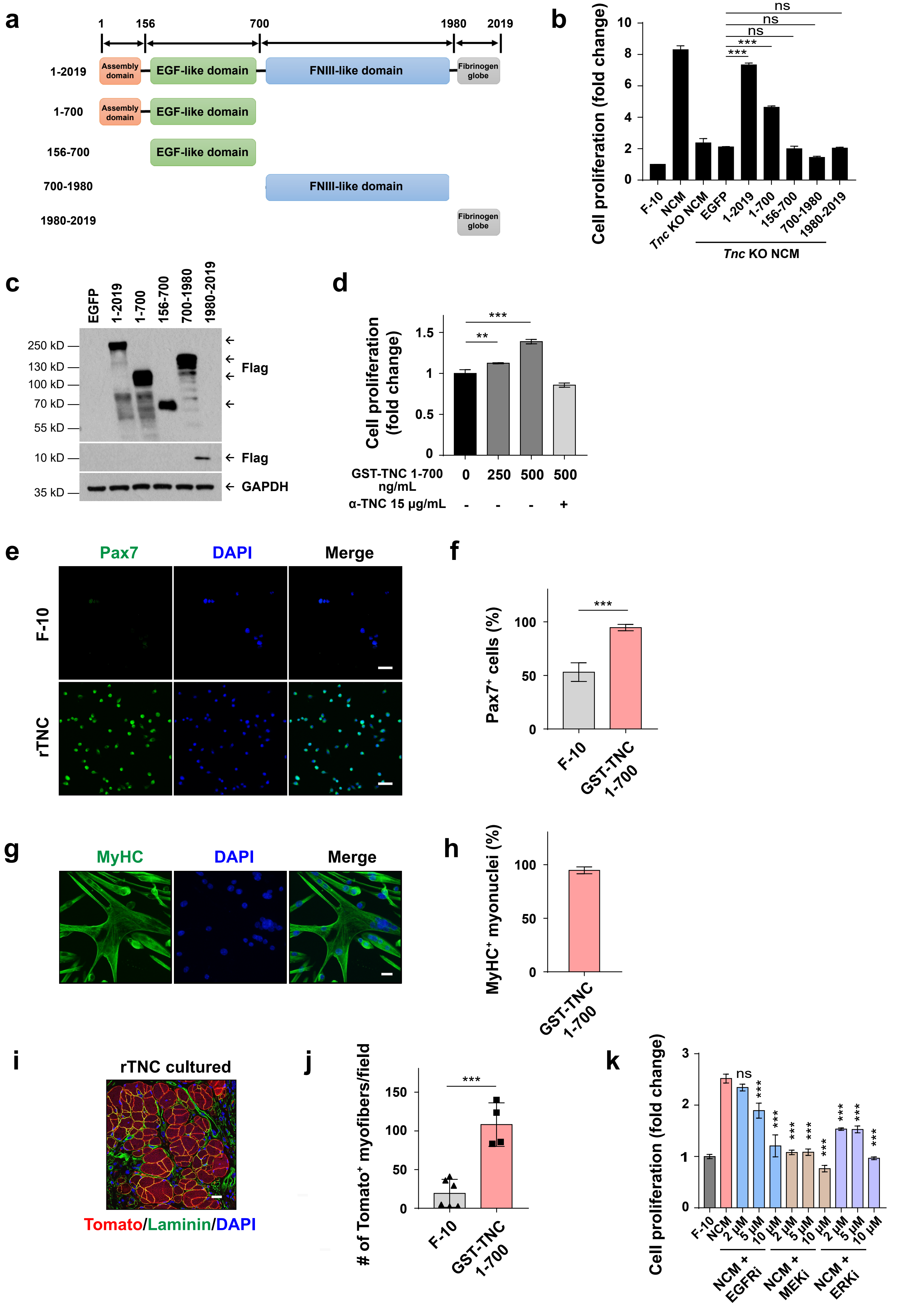

Zhou *et al.*, Figure S5

Supplementary information, Fig S5. | Recombinant TNC promotes MuSC proliferation.

a Domain structures and truncations of mouse Tenascin-C.

b Quantification of MuSCs cultured in NCM derived from truncated TNC expressing cells. *Tnc* knockout C2C12-*Mlkl*-TetON cells were rescued with re-expressing full length TNC or truncated TNC as shown in **a**. MuSCs were cultured for 3 passages followed by cell proliferation analysis, which was determined by measuring intracellular ATP levels. The data are expressed as the mean ± SD of 3 technical repeats.

c Immunoblotting analysis the expression of 3xFlag-HA-tagged full length and truncated TNC expression in *Tnc* knock out C2C12-*Mlkl*-TetON cells. TNC was re-expressed in knockout cells by retrovirus infection. GAPDH serves as the loading control.

d Quantification of MuSCs cultured in F-10 medium supplemented with GST-tagged TNC (1-700aa). Combinatory treatment with 15 μg/mL anti-TNC antibody was used to neutralize TNC, which serves as negative control. MuSCs were cultured for 3 passages followed by cell proliferation analysis, which was determined by measuring intracellular ATP levels. The data are expressed as the mean ± SD of 3 technical repeats.

e Immunofluorescence staining of Pax7 (green) in recombinant TNC-expanded MuSCs. F-10 medium was supplemented with 500 ng/mL GST-tagged TNC (1-700aa). Cells were cultured and expanded for 2 passages in the corresponding medium followed by immunofluorescent staining as described in the Methods. Nuclei were identified by staining with DAPI. Scale bars: 50 μm.

f Quantification of the $Pax7^+$ MuSCs as shown in **e**. Histogram represents the number of $Pax7^+$ cells out of 300 cells in each group. The data are expressed as the mean ± SD of 2 independent experiments.

g Representative immunofluorescence staining of MyHC (green) in differentiated MuSCs. MuSCs were expanded in F-10 medium supplemented with recombinant TNC (1-700aa, 500 ng/mL) for 2 passages and then cultured in differentiation medium for 48 hours. Nuclei were identified by staining with DAPI. Scale bar: 50 μm.

h Quantification of myonuclei within the $MyHC^+$ myofibers as shown in **g**. Histogram represents the percentage of myonuclei within the $MyHC^+$ myofibers out of 100 total myonucleus. The data are expressed as the mean ± SD of 2 independent experiments.

i Representative immunofluorescence staining of Laminin (green) merged with red fluorescent engrafted transplanted MuSCs. Red fluorescent MuSCs isolated from *R26*$^{mT/mG}$ transgenic mice were expanded in F-10 medium with or without recombinant TNC (GST-tagged TNC 1-700aa, 500 ng/mL) for 2 passages and then transplanted into X-ray irradiated recipient, the injured nonfluorescent *Rag2*$^{-/-}$;*Il2rg*$^{-/-}$ TA muscles. Experiments were performed in parallel and the same control group data (F-10 group) were shared here with as shown in Figure **3j**. Cross-sections of TA muscles were harvested at 28 days after transplantation and prepared for immunofluorescence staining of Laminin. Nuclei were identified by staining with DAPI. Scale bars: 25 µm.

j Quantification of the engrafted Tomato^{+} myofibers as shown in **i**. The number of Tomato^{+} myofibers from 24 fields (Leica SP8 microscopy with 20x objective magnification per field) were quantified for each mouse. Each dot represents an individual mouse. The data are expressed as the mean ± SD. $n = 6$ for the recipient mice of F-10 cultured MuSCs; $n = 4$ for the recipient mice of recombinant TNC (rTNC) cultured MuSCs.

k Quantification of MuSCs that were cultured in NCM with EGFR-MAPK signaling inhibitors. MuSCs were cultured for 48 hours. Cell proliferation was determined by measuring intracellular ATP levels using CellTiter-Glo assay. The data are expressed as the mean ± SD of 3 technical repeats. EGFRi: Afatinib; MEKi: Binimetinib; ERKi: SCH772984. *P* values were determined by one-way ANOVA with Tukey's multiple comparisons test, compared to the NCM group.

P values for **b** and **d** were determined by one-way ANOVA with Tukey's multiple comparisons test; *P* values for **f** and **j** were determined by unpaired two-tailed *t*-test; ns, non-significant; ** $P < 0.01$, *** $P < 0.005$.
