# Supplementary material for: Myofiber necroptosis promotes muscle stem cell proliferation via releasing Tenascin-C during regeneration
Source: Cell Res. 2020 Aug 24;30(12):1063–77. doi: 10.1038/s41422-020-00393-6 (PMC7784988; doi:10.1038/s41422-020-00393-6)
Supplement: Supplementary file 6 — Supplementary information, Fig. S6 [file 41422_2020_393_MOESM6_ESM.pdf]

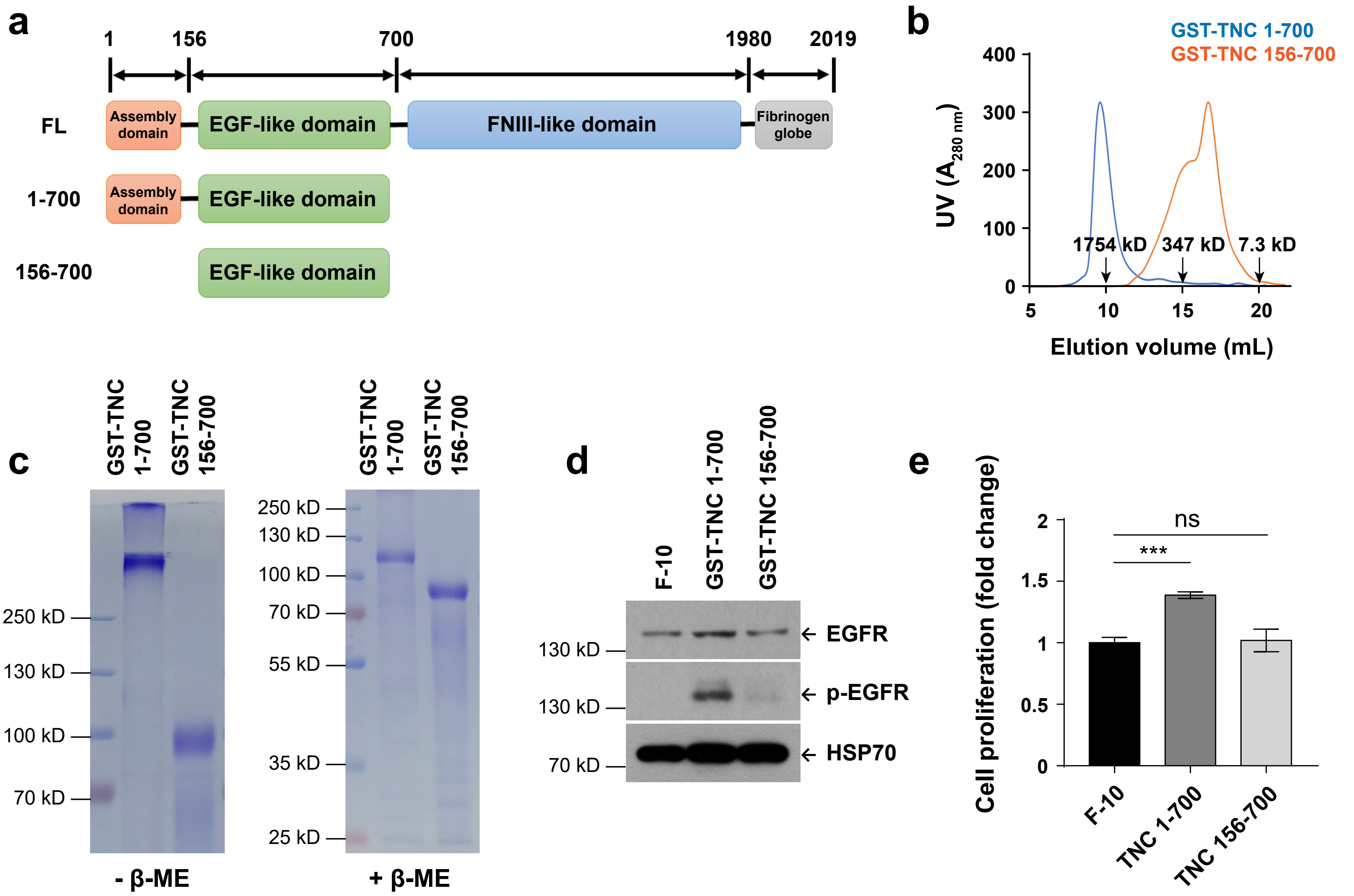

Zhou *et al.*, Figure S6

Supplementary information, Fig S6. | The assembly domain combining EGF-like domain of TNC are required for EGFR activation in MuSCs.

a Domain structures and truncations of mouse Tenascin-C.

b Size exclusion chromatography analysis of recombinant GST-tagged TNC 1-700aa and 156-700aa truncations.

c Coomassie blue staining of purified GST-tagged TNC 1-700aa and 156-700aa truncations with or without β-mercaptoethanol (β-ME) on SDS-PAGE.

d Immunoblotting analysis of EGFR activation in MuSCs cultured in F-10 medium or F-10 medium supplemented with recombinant TNC (500 ng/mL GST-tagged TNC 1-700aa and 156-700aa truncations). MuSCs cultured in the F-10 medium were treated with recombinant TNC for 4 hours and the equal whole cell lysates were subjected for SDS-PAGE.

e Quantification of MuSCs cultured in F-10 medium supplemented with GST-tagged TNC (1-700aa or 156-700aa). MuSCs were cultured for 3 passages followed by cell proliferation analysis, which was determined by measuring intracellular ATP levels. The data are expressed as the mean ± SD of 3 technical repeats. *P* values were determined by one-way ANOVA with Tukey's multiple comparisons test. ns, non-significant; *** $P < 0.005$.
