# Supplementary material for: Myofiber necroptosis promotes muscle stem cell proliferation via releasing Tenascin-C during regeneration
Source: Cell Res. 2020 Aug 24;30(12):1063–77. doi: 10.1038/s41422-020-00393-6 (PMC7784988; doi:10.1038/s41422-020-00393-6)
Supplement: Supplementary file 7 — Supplementary information, Fig. S7 [file 41422_2020_393_MOESM7_ESM.pdf]

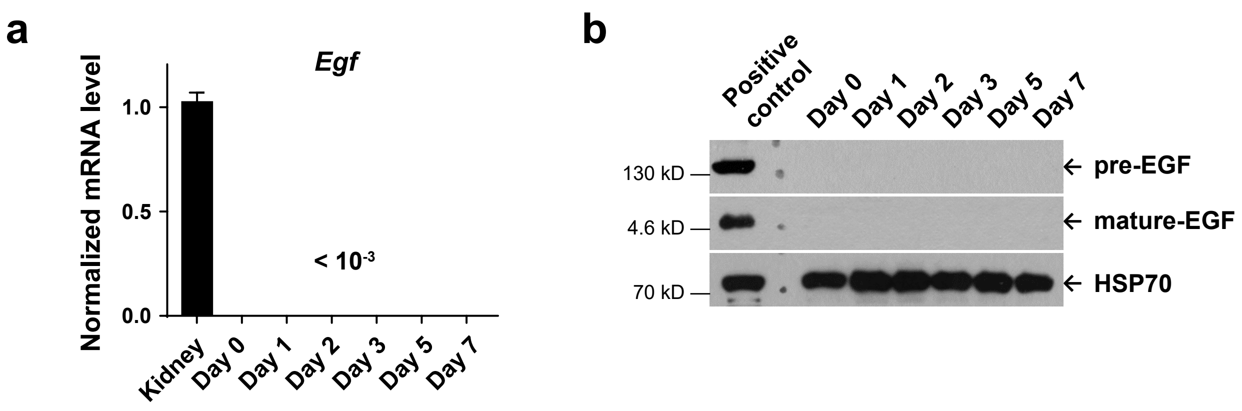

Zhou *et al.*, Figure S7

Supplementary information, Fig S7. | EGF is not detected in skeletal muscle before or after injury.

a qRT-PCR analysis of *Egf* mRNA level in TA muscles isolated from injured mice at the indicated days after CTX injection. The mRNA level of *Gapdh* was used as the internal control. Total mRNA extracted from adult mouse kidney was used as positive control. TA muscle samples isolated from 2 mice were pooled together in each group for qRT-PCR analysis. The data are expressed as the mean ± SD.

b Immunoblotting analysis of the expression of the pre- and mature-forms of EGF in whole-TA lysates of WT mice at the indicated days after CTX injection. TA muscle samples from 3 mice were pooled together per group. Adult mouse kidney was used as positive control for pre-EGF, and recombinant EGF was used as positive control for mature-EGF. HSP70 serves as the loading control. Experiments were repeated independently for three times.
