# Supplementary material for: Myofiber necroptosis promotes muscle stem cell proliferation via releasing Tenascin-C during regeneration
Source: Cell Res. 2020 Aug 24;30(12):1063–77. doi: 10.1038/s41422-020-00393-6 (PMC7784988; doi:10.1038/s41422-020-00393-6)
Supplement: Supplementary file 8 — Supplementary information, Table S1 [file 41422_2020_393_MOESM8_ESM.pdf]

Supplementary Table 1. List of Mass Spectrum Hits

Accession number	Protein description	Mascot score	MW	Matched queries	Matched peptides
IPI00123181	Myh9 Myosin-9	5839	226232	173	80
IPI00663627	Flnb Filamin-B	3878	277651	100	59
IPI00329872	Col1a1 Isoform 1 of Collagen alpha-1(I) chain	3871	137948	86	43
IPI00131138	Flna Isoform 1 of Filamin-A	3275	281046	97	50
IPI00129571	Col3a1 Collagen alpha-1(III) chain	2294	138858	54	26
IPI00877197	Col6a3 Uncharacterized protein	1776	185615	53	28
IPI00403938	Tnc Isoform 1 of Tenascin	1586	231659	42	24
IPI00664670	Flnc Uncharacterized protein	1405	292163	37	23
IPI00352163	Fn1 Putative uncharacterized protein	887	252843	21	12
IPI00830749	Col6a3 Uncharacterized protein (Fragment)	808	101633	24	14
IPI00465786	Tln1 Talin-1	771	269653	18	10
IPI00229509	Plec plectin isoform 1b2alpha	531	517663	13	8
IPI00121120	Col5a2 Collagen alpha-2(V) chain	498	144929	13	7
IPI00554929	Hsp90ab1 Heat shock protein HSP 90-beta	417	83229	13	7
IPI00454052	A2m Alpha-2-macroglobulin-P	330	164248	14	4
