# Supplementary material for: Myofiber necroptosis promotes muscle stem cell proliferation via releasing Tenascin-C during regeneration
Source: Cell Res. 2020 Aug 24;30(12):1063–77. doi: 10.1038/s41422-020-00393-6 (PMC7784988; doi:10.1038/s41422-020-00393-6)
Supplement: Supplementary file 9 — Supplementary information, Table S2 [file 41422_2020_393_MOESM9_ESM.pdf]

Supplementary Table 2. Primers for qPCR

Official Symbol	Official Full Name	Gene ID	Forward (5'-3')	Reverse (5'-3')
Ccnd1	cyclin D1	12443	GCGTACCCTGACACCAATCTC	CTCCTCTTCGCACTTCTGCTC
Egf	epidermal growth factor	13645	AGCATCTCTCGGATTGACCCA	CCTGTCCCGTTAAGGAAAACTCT
Gapdh	glyceraldehyde-3-phosphate dehydrogenase	14433	AGGTCGGTGTGAACGGATTTG	TGTAGACCATGTAGTTGAGGTCA
Mlkl	mixed lineage kinase domain-like	74568	AATTGTACTCTGGGAAATTGCCA	TCTCCAAGATTCCGTCCACAG
Tnc	tenascin C	21923	ACGGCTACCACAGAAGCTG	ATGGCTGTTGTTGCTATGGCA
Ptprc	protein tyrosine phosphatase, receptor type, C	19264	GTTTTCGCTACATGACTGCACA	AGGTTGTCCAACTGACATCTTTC

Primers were acquired from validation results of PrimerBank (https://pga.mgh.harvard.edu/primerbank/).
