# Supplementary material for: Myofiber necroptosis promotes muscle stem cell proliferation via releasing Tenascin-C during regeneration
Source: Cell Res. 2020 Aug 24;30(12):1063–77. doi: 10.1038/s41422-020-00393-6 (PMC7784988; doi:10.1038/s41422-020-00393-6)
Supplement: Supplementary file 10 — Supplementary information, Table S3 [file 41422_2020_393_MOESM10_ESM.pdf]

Supplementary Table 3. Primers of sgRNA

Primer sequences for in vitro Knock-out.

Gene	Gene ID	Sence (5'-3')	Anti-Sence (5'-3')
sg-control	\	CACCGGGCCACGAGTTCGAGATCGA	AAACTCGATCTCGAACTCGTGGCCC
sg-Tnc-1	21923	CACCGGTCATTGCAGTCGTTCGGAC	AAACGTCCGAACGACTGCAATGACC
sg-Tnc-2		CACCGACCGCGGTCGAAGCAGTCGT	AAACACGACTGCTTCGACCGCGGTC
sg-Tnc-3		CACCGCGCGGTCGAAGCAGTCGTTG	AAACCAACGACTGCTTCGACCGCGC
sg-Tnc-4		CACCGATCCACCACCATTCGGGAGC	AAACGCTCCCGAATGGTGGTGGATC

sgRNA sequences were acquired from high ranked hits predicted by Feng Zhang's lab (http://crispor.tefor.net/).

Primer sequences for AAV mediated in vivo Knock-out.

Gene	Gene ID	Sence (5'-3')	Anti-Sence (5'-3')
sg-control	\	CACCGCACTACCAGAGCTAACTCA	AAACTGAGTTAGCTCTGGTAGTGC
sg-Egfr-1	13649	CACCGACCGCGAGAACCACACTGC	AAACGCAGTGTGGTTCTCGCGGTC
sg-Egfr-2		CACCGTGGTTCTCGCGGTCCCTGA	AAACTCAGGGACCGCGAGAACCAC
sg-Egfr-3		CACCGTTCCTCCAACGCCCCACCTG	AAACCAGGTGGGGCGTTGGAGGAAC

sgRNA sequences were acquired from high ranked hits predicted by CCTop (https://crispr.cos.uni-heidelberg.de/).
